# Genetic and Functional Analyses of *SHANK2* Mutations Suggest a Multiple Hit Model of Autism Spectrum Disorders

**DOI:** 10.1371/journal.pgen.1002521

**Published:** 2012-02-09

**Authors:** Claire S. Leblond, Jutta Heinrich, Richard Delorme, Christian Proepper, Catalina Betancur, Guillaume Huguet, Marina Konyukh, Pauline Chaste, Elodie Ey, Maria Rastam, Henrik Anckarsäter, Gudrun Nygren, I. Carina Gillberg, Jonas Melke, Roberto Toro, Beatrice Regnault, Fabien Fauchereau, Oriane Mercati, Nathalie Lemière, David Skuse, Martin Poot, Richard Holt, Anthony P. Monaco, Irma Järvelä, Katri Kantojärvi, Raija Vanhala, Sarah Curran, David A. Collier, Patrick Bolton, Andreas Chiocchetti, Sabine M. Klauck, Fritz Poustka, Christine M. Freitag, Regina Waltes, Marnie Kopp, Eftichia Duketis, Elena Bacchelli, Fiorella Minopoli, Liliana Ruta, Agatino Battaglia, Luigi Mazzone, Elena Maestrini, Ana F. Sequeira, Barbara Oliveira, Astrid Vicente, Guiomar Oliveira, Dalila Pinto, Stephen W. Scherer, Diana Zelenika, Marc Delepine, Mark Lathrop, Dominique Bonneau, Vincent Guinchat, Françoise Devillard, Brigitte Assouline, Marie-Christine Mouren, Marion Leboyer, Christopher Gillberg, Tobias M. Boeckers, Thomas Bourgeron

**Affiliations:** 1 Human Genetics and Cognitive Functions, Institut Pasteur, Paris, France; 2 CNRS URA 2182 “Genes, synapses and cognition,” Institut Pasteur, Paris, France; 3 University Denis Diderot Paris 7, Paris, France; 4 Institute of Anatomy and Cell Biology, Ulm University, Ulm, Germany; 5 Assistance Publique-Hôpitaux de Paris, Robert Debré Hospital, Department of Child and Adolescent Psychiatry, Paris, France; 6 INSERM, U952, Paris, France; 7 CNRS, UMR 7224, Paris, France; 8 UPMC Univ Paris 06, Paris, France; 9 Department of Clinical Sciences in Lund, Lund University, Lund, Sweden; 10 Institute of Clinical Sciences, Lund University, Malmö, Sweden; 11 Gillberg Neuropsychiatry Centre, University of Gothenburg, Göteborg, Sweden; 12 Institute of Neuroscience and Physiology, Department of Pharmacology, Gothenburg University, Göteborg, Sweden; 13 Eukaryote Genotyping Platform, Genopole, Institut Pasteur, Paris, France; 14 Behavioural and Brain Sciences Unit, Institute of Child Health, University College London, London, United Kingdom; 15 Department of Medical Genetics, University Medical Center Utrecht, Utrecht, The Netherlands; 16 Wellcome Trust Centre for Human Genetics, University of Oxford, Oxford, United Kingdom; 17 Department of Medical Genetics, University of Helsinki, Helsinki, Finland; 18 Academic Department of Child and Adolescent Psychiatry, Institute of Psychiatry, King's College London, London, United Kingdom; 19 Social Genetic Developmental Psychiatry Centre, Institute of Psychiatry, King's College London, London, United Kingdom; 20 Division of Molecular Genome Analysis, German Cancer Research Center (DKFZ), Heidelberg, Germany; 21 Department of Child and Adolescent Psychiatry, Psychosomatics and Psychotherapy, Goethe University, Frankfurt am Main, Germany; 22 Department of Biology, University of Bologna, Bologna, Italy; 23 Division of Child Neurology and Psychiatry, Department of Paediatrics, University of Catania, Catania, Italy; 24 Stella Maris Clinical Research Institute for Child and Adolescent Neuropsychiatry, Pisa, Italy; 25 Division of Child Neurology and Psychiatry, Department of Pediatrics, University of Catania, Catania, Italy; 26 Instituto Nacional de Saude Dr Ricardo Jorge, Lisbon, Portugal; 27 Instituto Gulbenkian de Ciencia, Oeiras, Portugal; 28 Center for Biodiversity, Functional and Integrative Genomics, Faculdade de Ciências da Universidade de Lisboa, Lisboa, Portugal; 29 Unidade Neurodesenvolvimento e Autismo, Centro Investigação e Formação Clinica, Hospital Pediátrico Coimbra e Faculdade Medicina, Universidade Coimbra, Coimbra, Portugal; 30 The Centre for Applied Genomics and Program in Genetics and Genomic Biology, The Hospital for Sick Children, Toronto, Canada; 31 Centre National de Génotypage, Evry, France; 32 INSERM U771 and CNRS 6214, Angers, France; 33 Département de Biochimie et Génétique, Centre Hospitalier Universitaire, Angers, France; 34 CADIPA–Centre de Ressources Autisme Rhône-Alpes, Saint Egrève, France; 35 Genetics Department, Hôpital Couple-Enfant, Grenoble, France; 36 INSERM, U955, Psychiatrie Génétique, Créteil, France; 37 Université Paris Est, Faculté de Médecine, Créteil, France; 38 AP-HP, Hôpital H. Mondor–A. Chenevier, Département de Psychiatrie, Créteil, France; 39 Institute of Child Health, University College London, London, United Kingdom; Yale University School of Medicine, United States of America

## Abstract

Autism spectrum disorders (ASD) are a heterogeneous group of neurodevelopmental disorders with a complex inheritance pattern. While many rare variants in synaptic proteins have been identified in patients with ASD, little is known about their effects at the synapse and their interactions with other genetic variations. Here, following the discovery of two *de novo SHANK2* deletions by the Autism Genome Project, we identified a novel 421 kb *de novo SHANK2* deletion in a patient with autism. We then sequenced *SHANK2* in 455 patients with ASD and 431 controls and integrated these results with those reported by Berkel *et al.* 2010 (n = 396 patients and n = 659 controls). We observed a significant enrichment of variants affecting conserved amino acids in 29 of 851 (3.4%) patients and in 16 of 1,090 (1.5%) controls (P = 0.004, OR = 2.37, 95% CI = 1.23–4.70). In neuronal cell cultures, the variants identified in patients were associated with a reduced synaptic density at dendrites compared to the variants only detected in controls (P = 0.0013). Interestingly, the three patients with *de novo SHANK2* deletions also carried inherited CNVs at 15q11–q13 previously associated with neuropsychiatric disorders. In two cases, the nicotinic receptor *CHRNA7* was duplicated and in one case the synaptic translation repressor *CYFIP1* was deleted. These results strengthen the role of synaptic gene dysfunction in ASD but also highlight the presence of putative modifier genes, which is in keeping with the “multiple hit model” for ASD. A better knowledge of these genetic interactions will be necessary to understand the complex inheritance pattern of ASD.

## Author Summary

Autism spectrum disorders (ASD) are a heterogeneous group of neurodevelopmental disorders with a complex inheritance pattern. While mutations in several genes have been identified in patients with ASD, little is known about their effects on neuronal function and their interaction with other genetic variations. Using a combination of genetic and functional approaches, we identified novel *SHANK2* mutations including a *de novo* loss of one copy of the *SHANK2* gene in a patient with autism and several mutations observed in patients that reduced neuronal cell contacts *in vitro*. Further genomic analysis of three patients carrying *de novo SHANK2* deletions identified additional rare genomic imbalances previously associated with neuropsychiatric disorders. Taken together, these results strengthen the role of synaptic gene dysfunction in ASD but also highlight the presence of putative modifier genes, which is in keeping with the “multiple hit model” for ASD. A better knowledge of these genetic interactions will be necessary to understand the complex inheritance pattern of ASD.

## Introduction

Autism spectrum disorders (ASD) are characterized by impairments in reciprocal social communication and stereotyped behaviors [1]. The prevalence of ASD is about 1/100, but closer to 1/300 for typical autism [2]. ASD are more common in males than females, with a 4∶1 ratio. Previously, twin and family studies have conclusively described ASD as the most “genetic” of neuropsychiatric disorders, with concordance rates of 82–92% in monozygotic twins versus 1–10% in dizygotic twins [3], but a recent study finds evidence for a more substantial environmental component [4]. In the absence of Mendelian inheritance patterns, ASD were first considered to be polygenic, i.e., a disorder caused by multiple genetic risk factors, each of weak effect. More recently, an alternative model was proposed that considered ASD as a group of disorders caused by heterogeneous genetic risk factors influencing common neuronal pathways [5], [6]. It was supported by the identification of apparently monogenic forms of ASD, each affecting a limited number of patients (1–2% for the most replicated genes) [7]–[14]. In this model, eventually a single highly penetrant mutation would be sufficient to produce ASD. However, the occurrence of two or more deleterious copy number variants (CNV) or mutations in a subset of patients also suggested that independent loci could act in concert to induce the development of ASD [9], [13]–[16]. In line with these findings, the recent observation that patients with a deletion at 16p12.1 were more likely to carry an additional large CNV agrees with a “two-hit model” for developmental disorders [17].

The genetic causes of ASD are diverse [18], but the main category of genes associated with the disorder is related to the development and function of neuronal circuits [6], [19]. Mutations of genes coding for synaptic cell adhesion molecules and scaffolding proteins, such as neuroligins (NLGN), neurexins (NRXN) and SHANK, have been recurrently reported in patients with ASD [7]–[10], [13], [14], [20]. These proteins play a crucial role in the formation and stabilization of synapses [21], as well as in synaptic homeostasis [22]. *SHANK2* and *SHANK3* code for scaffolding proteins located in the postsynaptic density (PSD) of glutamatergic synapses. Deletions of *ProSAP2/SHANK3* at chromosome 22q13 are one of the major genetic abnormalities in neurodevelopmental disorders [20], and mutations of *ProSAP2/SHANK3* have been identified in patients with ASD, intellectual disability (ID) and schizophrenia [7], [23]–[25]. Mutations of *ProSAP1/SHANK2* have also recently been reported in both, ASD and ID [9], [26]. The difference in clinical outcome of mutation carriers has been attributed to the presence of still uncharacterized additional genetic, epigenetic and/or environmental factors [27].

In order to better understand the role of the NRXN-NLGN-SHANK pathway in ASD, we first aimed to describe *SHANK2* isoform expression in different tissues of healthy individuals. To investigate the role of this pathway in ASD, we screened for *SHANK2* CNVs and coding mutations in a large sample of patients with ASD and controls. We provide genetic and functional evidence that *SHANK2* is associated with ASD, and that its mutations affect the number of synapses. Additionally, we report the co-occurrence of *SHANK2 de novo* deletions and inherited CNVs altering neuronal genes, suggesting that epistasis between specific loci in the genome could modulate the risk for ASD.

## Results

### *SHANK2* isoforms are differentially expressed in human tissues

**Figure 1:**
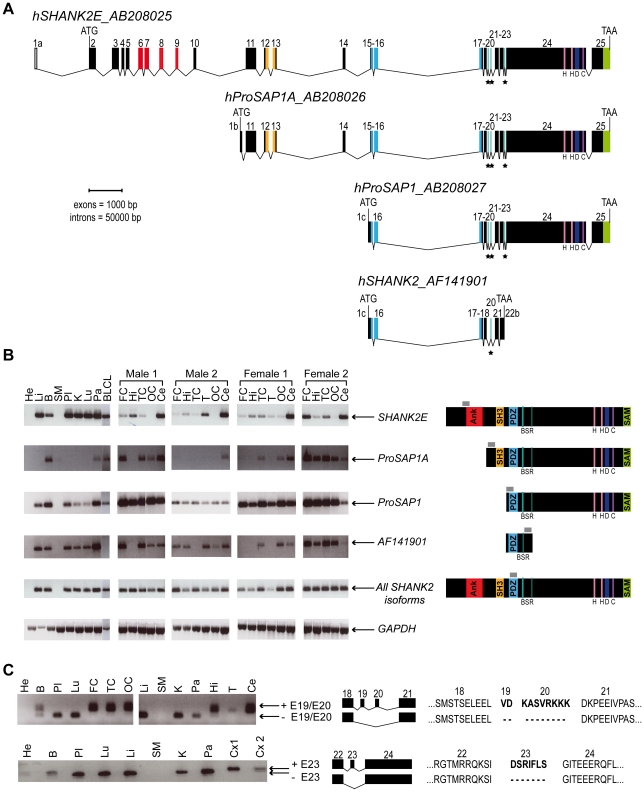
Genomic structure, isoforms, and expression of human *SHANK2*. A. Genomic structure of the human *SHANK2* gene. Transcription of *SHANK2* produces four main mRNA from three distinct promoters: *SHANK2E* (*AB208025*), *ProSAP1A* (*AB208026*), *ProSAP1* (*AB208027*) and *AF141901*. There are three translation starts: in exon 2 for *SHANK2E*, in exon1b for *ProSAP1A*, and in exon1c for *ProSAP1* and *AF141901*; and two independent stop codons: in exon 22b for *AF141901* and in exon 25 for *SHANK2E*, *ProSAP1A* and *ProSAP1*. Conserved domains of protein interaction or protein binding site are represented in color: ANK (red), SH3 (orange), PDZ (blue) and SAM (green), H (pink), D, (dark blue) and C (purple). Black stars identify the alternative spliced exons (‘brain-specific exons’ in turquoise: 19, 20 and 23). B. RT-PCRs of *SHANK2* isoforms on RNA from different human control tissues (Clontech), and different brain regions of four controls (2 males and 2 females). The amplified regions specific to each isoform of *SHANK2* are indicated by gray boxes. C. Alternative splicing of human *SHANK2*; exons 19, 20 and 23 are specific to the brain. ANK, ankyrin; SH3, Src homology 3; PDZ, PSD95/DLG/ZO1; SAM, sterile alpha motif; He, heart; Li, liver; B, brain; SM, skeletal muscle; Pl, placenta; K, kidney; Lu, lung; Pa, pancreas; FC, frontal cortex; Hi, hippocampus; TC, temporal cortex; T, thalamus; OC, occipital cortex; Ce, cerebellum; Cx, whole cortex; BLCL, B lymphoblastoid cell lines; GAPDH, glyceraldehyde 3-phosphate dehydrogenase; BSR, brain specific region; H, homer binding site; D, dynamin binding site; C, cortactin binding site. The ages of the two males and the two females studied were 74, 42, 55, and 36 years with a post-mortem interval of 10, 21, 24, and 2 h, respectively.

In order to characterize all isoforms of *SHANK2*, we scanned genomic databases for specific Expressed Sequence Tags (ESTs) and spliced isoforms. The human *SHANK2* gene (*NM_012309.3*) spans 621.8 kb and contains 25 exons (Figure 1). The longest *SHANK2* isoform (*SHANK2E*, *AB208025*) contains ankyrin (ANK) repeats at the N-terminus, followed by a Src homology 3 (SH3) domain, a PSD95/DLG/ZO1 (PDZ) domain, a proline-rich region and a sterile alpha motif (SAM) domain at its C-terminus region. All these domains are involved in protein-protein interactions that bridge glutamate receptors, scaffolding proteins and intracellular effectors to the actin cytoskeleton [28], [29]. Two additional isoforms, *ProSAP1A* (*AB208026*) and *ProSAP1* (*AB208027*), originating from distinct promoters, were previously detected in the rat [30], [31]. Finally, the shortest isoform (*AF141901*), also originally described in the rat, results in premature termination of the transcription before the SAM domain due to an alternative 3′ end in exon 22 [32] (Figure 1A). To validate these *SHANK2* isoforms in humans, we used specific RT-PCRs and sequencing (Figure 1B). Almost all tissues tested (brain, liver, placenta, kidney, lung, pancreas and lymphoblastoid cell lines) expressed *SHANK2* mRNA, except heart and skeletal muscle, for which no expression was detected. We observed inter-individual differences in the relative amount of *SHANK2* mRNA that were confirmed by using independent RT-PCRs and primers (not shown). Such differences have been previously reported for other synaptic genes such as *NLGN1-4Y*, *PCHD11X/Y*, and *SHANK3* [7], [8], [33] and might be the consequence of polymorphisms located in specific regulatory sequences and/or activity dependent expression of this family of post-synaptic proteins [34]. Notably, exons 19, 20 and 23 were found to be expressed only in brain in all individuals tested (Figure 1C). Such brain specific splicing has been already observed for exon 18 in *SHANK3* [7], which is similar to exon 19 and 20 in *SHANK2*. These ‘brain-specific exons’ code for a region in SHANK2/3 located between the PDZ and the proline rich domains. Finally, in contrast to previous results [26], we detected the longest *SHANK2E* isoform in all independent samples of human brain, with high expression in the cerebellum (Figure 1, Figure S1). This *Shank2E* isoform was also expressed in the cerebellum and in the liver of rat embryo at E19 (Figure S1).

### A *de novo* deletion of *SHANK2* in a patient with ASD

**Figure 2:**
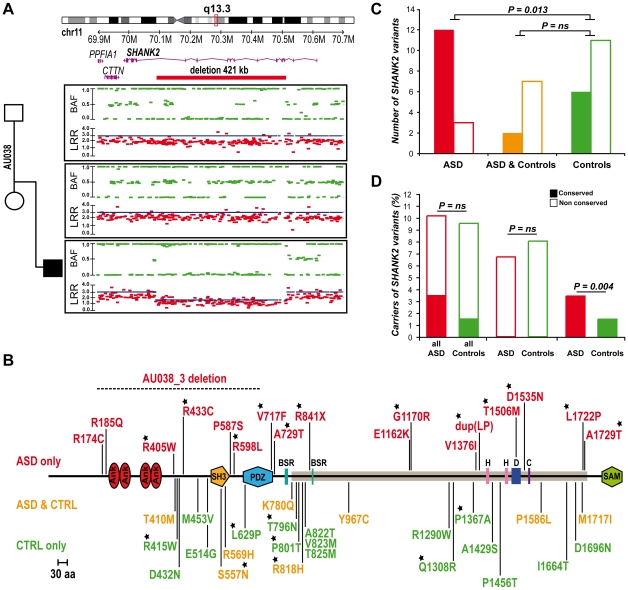
*SHANK2* mutations in patients with ASD. A. A heterozygous deletion of *SHANK2* was identified with the Illumina Human 1M-Duo SNP array in a patient with autism (AU038_3). The deletion spans 421 kb on chromosome 11q13.3, covers twelve exons of the human *SHANK2* and is not present in the parents. Each dot shows Log R Ratio (LRR; in red) and B allele frequency (BAF; in green). QuantiSNP score is represented with a blue line and indicates the deletion size. B. Location of the CNV and sequence variants (from this study and Berkel *et al.* 2010) along the SHANK2 protein: in red the variations specific to ASD, in orange the variations shared by ASD and controls and in green the variations specific to controls [26]. The breakpoints of the *SHANK2* deletion in AU038_3 are represented with a dotted line on the protein. Stars indicate the variants affecting conserved amino acids. C. A total of 40 variants were identified and variants affecting conserved amino acids in other SHANK proteins are enriched in patients with ASD (n_conserved_ = 12 and n_non-conserved_ = 3) compared with controls (n_conserved_ = 6 and n_non-conserved_ = 11) (Fisher's exact test 1-sided, P = 0.013, OR = 6.83, 95% IC = 1.19–53.40). D. The percentage of carriers of *SHANK2* variants in patients with ASD and Controls. Variants affecting a conserved amino acid among the SHANK proteins are enriched in patients with ASD (n_conserved_ = 29 and n_non-conserved_ = 822) compared with controls (n_conserved_ = 16 and n_non-conserved_ = 1074) (Fisher's exact test 1-sided, P = 0.004, OR = 2.37, 95% CI = 1.23–4.70). Open squares and filled squares represent the non-conserved and conserved amino acids, respectively. ANK, Ankyrin repeat domain; SH3, Src homology 3 domain; PDZ, postsynaptic density 95/Discs large/zona occludens-1 homology domain; SAM, sterile alpha motif domain; BSR, brain specific region; H, homer binding site; D, dynamin binding site; C, cortactin binding site. The proline-rich region is represented as a horizontal gray line.

Berkel *et al.* 2010 recently identified two independent *de novo SHANK2* deletions in two patients, one with ID and another one with ASD [26]. In addition, whole genome analysis performed by the Autism Genome Project (AGP) using Illumina 1M single nucleotide polymorphism (SNP) arrays detected one additional *de novo SHANK2* deletion in a patient (6319_3) with ASD [9] (the second patient described by the AGP, 5237_3, is patient SK0217-003 reported in Berkel *et al.* 2010 [26]). Recently, a 3.4 Mb de novo deletion including *SHANK2* was observed in a female patient with speech and developmental delay [35]. To follow up on these results, we genotyped an independent sample of 260 patients with ASD using Illumina 1M Duo SNP arrays (Table S1). In this sample, we detected a 421.2 kb deletion within *SHANK2* in patient AU038_3 with autism and moderate ID (see patient section in Materials and Methods, and Table S2). The deletion covered twelve exons (E5–E16) and altered all *SHANK2* isoforms (Figure 2A). No other deleterious variants in the remaining copy of *SHANK2* were detected by sequencing. The parents did not carry the deletion, indicating a *de novo* event. The deletion was validated by quantitative PCR analysis using DNA from an independent blood sample from all members of the family and SNP analysis indicated that the deletion originated on the maternal chromosome (Figure S2). *SHANK2* deletions were absent in more than 5000 controls [9], [26] and not listed in the Database of Genomic Variants (DGV; http://projects.tcag.ca/variation/).

### *SHANK2* coding variants affecting conserved amino acids are enriched in patients with ASD

To probe for additional mutations, we first sequenced all exons of the longest *SHANK2E* isoform in 230 patients with ASD and 230 controls. We then sequenced an additional sample of 225 patients and 201 controls (Table S1) for the *ProSAP1A* isoform that corresponds to the major *SHANK2* isoform in the brain. Since we screened all *SHANK2* isoforms, we used a nomenclature including the *SHANK2E* isoform that differed from Berkel *et al.* 2010 [26]. Within the 9 coding exons specific to *SHANK2E*, we identified R174C (rs7926203) listed in dbSNP in 2 independent patients with ASD and R185Q in one patient with ASD. For this isoform, no variant was identified in the control sample. Within the *ProsSAP1A* isoform, we identified 24 non-synonymous variations. When these results are integrated with those obtained by Berkel *et al.* 2010, a total of 40 variants of *ProsSAP1A* including 3 already reported in dbSNP were identified (Figure 2B, Table 1, Figure S3). Only two variants (Y967C and R569H) with MAF>1% are detected and there is no enrichment of rare variants of *SHANK2* (MAF<1%) in patients with ASD compared with controls. Because variants affecting conserved amino acids in the SHANK proteins are most likely to have a functional effect, we tested whether there was an enrichment of these variants in patients compared to controls. The alignment of the SHANK protein sequences and the conservation of the variants are indicated in the Table S5. In both mutation screening studies, the first performed by Berkel *et al.* 2010 and the second presented here, we observed an enrichment of variants affecting conserved amino acids in patients compared with controls (Figure 2C, Table S5 and Table S7). Overall, 12 of 15 (80%) of the variants identified only in the patient sample affected conserved amino acids compared with only 6 of 17 (35.3%) in controls (Fisher's exact test 1-sided, P = 0.013, OR = 6.83, 95% IC = 1.19–53.40). Because several independent patients carried these variants (Table 1), the enrichment is even more significant when the number of carriers was considered. The variants affecting conserved amino acids were observed in 29 of 851 (3.4%) patients and in 16 of 1090 (1.5%) controls (Fisher's exact test 1-sided, P = 0.004, OR = 2.37, 95% CI = 1.23–4.70). A total of 8 variants were identified in patients and controls. Among these 8 variants, 2 affected conserved amino acids (R818H and S557N). The variant S557N was observed in 9 of 851 (1.06%) independent families with ASD and in 3 of 1090 (0.28%) controls (Fisher's Exact Test one sided, P = 0.029, OR = 3.87; 95% CI = 0.96–22.29). It affects a conserved serine with a high probability of being phosphorylated and located in the SH3 domain of all SHANK proteins. This domain binds to GRIP and b-PIX, two proteins linking SHANK to glutamate AMPA receptors and actin skeleton, respectively [36]. In our initial mutation screen, R818H was observed in 5 of 230 patients with ASD and 0 of 230 controls. In order to determine if R818H was more frequent in the patients with ASD, we screened an additional sample of 3020 individuals with ASD, 1783 controls from European descent, and the Human Genome Diversity Panel (HGDP) control dataset (Table S3 and S4). R818H was virtually absent outside Europe and had the highest allelic frequency (2.37%) in Finland, but overall its frequency was not higher in patients with ASD compared with controls (ASD 32/3250 (1.0%); controls 27/2030 (1.33%); Fisher's exact test 2-sided, P = 0.28) (Table S3).

**Table 1:** *ProSAP1A/SHANK2* variations identified in 851 patients with ASD and 1,090 controls.

	Detected variants			Conservation in SHANK proteins^d^	Frequency		Study
	Exon	Nucleotide/dbSNP^a^	Amino acid		ASD (n = 851)	Controls (n = 1090)	
ASD only	E11	G70344397A	R405W	Yes (S1 & S3)	1	0	Berkel *et al.* 2010
	E11	G70344284A	R443C	Yes (S3)	1	0	This study
	E13	G70322214A	P587S	No	1	0	Berkel *et al.* 2010
	E14	C70222501A	R598L	Yes (S3)	1	0	This study
	E17	C70026597A	V717F^b^	Yes (S1 & S3)	1	0	This study
	E17	C70026561T	A729T^b^	Yes (S3)	1	0	This study
	E22	G70014059A	R841X	Yes (S3)	1	0	Berkel *et al.* 2010
	E24	C70010562T	E1162K	No	1	0	This study
	E24	C70010538T	G1170R^b^	Yes (S1 & S3)	1	0	This study
	E24	C70009920T	V1376I	No	1	0	This study
	E24	dup(AACGGT) 70009882–70009887	dup(LP) 1387–1388	Yes (S1 & S3; S1)	1	0	Berkel *et al.* 2010
	E24	G70009529A	T1506M	Yes (S1 & S3)	1	0	Berkel *et al.* 2010
	E24	C70009443T	D1535N^b^	Yes (S1 & S3)	1	0	This study
	E25	A69997007G	L1722P^b^	Yes (S1 & S3)	1	0	This study
	E25	C69996987T	A1729T	Yes (S3)	1	0	Berkel *et al.* 2010
ASD & Controls	E11	G70344381A	T410M	No	1	2	This study & Berkel *et al.* 2010
	E13	C70322303T	S557N	Yes (S1 & S3)	9	3	This study & Berkel *et al.* 2010
	E13	C70322267T	R569H	No	17	28	This study & Berkel *et al.* 2010
	E21	T70016189G rs55968949	K780Q	No	4	4	This study & Berkel *et al.* 2010
	E22	C70014127T rs117843717	R818H^b^^,^ c	Yes (S3)	8	7	This study & Berkel *et al.* 2010
	E24	T70011146C rs62622853	Y967C	No	27	38	This study & Berkel *et al.* 2010
	E24	G70009289A	P1586L	No	4	1	This study & Berkel *et al.* 2010
	E25	C69997021T	M1717I	No	2	2	This study & Berkel *et al.* 2010
Controls only	E11	G70344367A	R415W	Yes (S3)	0	1	Berkel *et al.* 2010
	E11	C70344316T	D432N	No	0	1	Berkel *et al.* 2010
	E11	T70344253C	M453V	No	0	1	This study
	E12	T70330877C	E514G	No	0	1	Berkel *et al.* 2010
	E15	A70185399G	L629P	Yes (S1 & S3)	0	1	This study
	E21	G70016140T	T796N	Yes (S3)	0	1	Berkel *et al.* 2010
	E21	G70016126T	P801T	Yes (S1 & S3)	0	1	Berkel *et al.* 2010
	E22	C70014116T	A822T	No	0	1	This study
	E22	C70014113T	V823M	No	0	1	This study
	E22	G70014106A	T825M	No	0	1	Berkel *et al.* 2010
	E24	G70010178A	R1290W	No	0	1	This study
	E24	T70010123C	Q1308R	Yes (S3)	0	1	This study
	E24	G70009947C	P1367A	Yes (S1 & S3)	0	1	This study
	E24	A70009759G	A1429S	No	0	2	Berkel *et al.* 2010
	E24	G70009680T	P1456T	No	0	1	Berkel *et al.* 2010
	E25	A69997181G	I1664T	No	0	2	Berkel *et al.* 2010
	E25	C69997086T	D1696N	No	0	1	Berkel *et al.* 2010
Total variants					87 (10.2%)	104 (9.5%)	Fisher's exact test 1-sided, P = 0.34, OR = 1.08, 95% CI = 0.79–1.47
Total variants with MAF<1%					43 (5.1%)	38 (3.5%)	Fisher's exact test 1-sided, P = 0.06, OR = 1.47, 95% CI = 0.92–2.37
Total conserved variants					29 (3.4%)	16 (1.5%)	Fisher's exact test 1-sided, P = 0.004, OR = 2.37, 95% CI = 1.23–4.70

a Nucleotide positions are according to *NM 012309.3* from NCBI36/hg18 on the positive DNA strand; The patients with ASD and the controls used for this analysis came from this study (455 ASD & 431 controls) and from the study of Berkel *et al.* 2010 (396 ASD & 659 controls);

b A screening of V717F, A729T, R818H, G1170R, D1535N and L1722P was performed in 948 subjects from the Human Genome Diversity Panel (V717F = 0/948; A729T = 0/948; R818H = 5/948; G1170R = 0/948; D1535N = 0/948; L1722P = 0/948);

c A screen of R818H was performed in additional patients and controls (ASD 32/3250 (1.0%); controls 27/2030 (1.33%); Fisher's exact test 2-sided, P = 0.28). Fisher's exact test was used for statistical analysis;

d “Yes” indicates if amino acid is conserved in SHANK1 (S1), SHANK3 (S3) or both (S1 & S3); MAF, Minor Allele Frequency.

**Figure 3:**
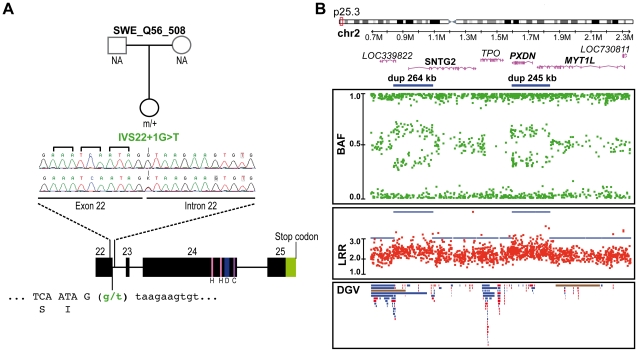
Genetic alterations identified in the control subject SWE_Q56_508. A. *SHANK2* splice mutation (IVS22+1G>T) detected in a Swedish female control, SWE_Q56_508. The mutation altered the donor splicing site of exon 22 and led to a premature stop in all *SHANK2* isoforms except for the *AF1411901* isoform, where it altered the protein sequence (G263V). B. CNVs in the same individual altering *LOC339822*, *SNTG2*, *PXDN* and *MYT1L*. The two close duplications span 264 kb and 245 kb on chromosome 2 and altered *LOC339822* and *SNTG2*, and *PXDN* and *MYT1L*, respectively. Dots show the B allele frequency (BAF; in green), Log R ratio (LRR; in red), and QuantiSNP score (in blue). Lower panel: all CNVs listed in the Database of Genomic Variants (DGV) are represented: loss (in red), gain (in blue), gain or loss (in brown). H, homer binding site; D, dynamin binding site; C, cortactin binding site.

Finally, and unexpectedly, during this additional mutation screening, we detected a variation (IVS22+1G>T) altering the consensus donor splice site of exon 22 in a Swedish control, SWE_Q56_508 (Figure 3A). This variant was predicted to disrupt all *SHANK2* isoforms by deleting the proline rich and the SAM domain, except for the shortest isoform *AF141901*, where the mutation is located in the open reading frame (ORF) and should lead to a G263V change. This variant was not observed in 1786 patients or 1407 controls, and is not listed in dbSNP. This control female was part of a previous epidemiological study [37] and had been extensively examined for anthropometrics and cardiovascular risk factors such as blood pressure and levels of all major hormones. In addition, she was ascertained for axis I psychiatric disorders and personality traits using the Temperament and Character Inventory (TCI) [38] and the Karolinska Scales of Personality (KSP) [39]. Notably, despite the predicted deleterious effect of the mutation, this subject had no major somatic or psychiatric health problems. Regarding personality traits, none of her scores for TCI items were different from those found in the general population. KSP assessment showed that her scores for neuroticism (51.3), nonconformity/aggressiveness (56.7), and psychoticism (50.5) were not different from the general population (mean ± SD = 50±10). However, she displayed a high score (61.4) for the extraversion factor, and for one of its subscales, monotony avoidance [40].

### Several *SHANK2* variants identified in patients alter synapse density in cultured neurons

**Figure 4:**
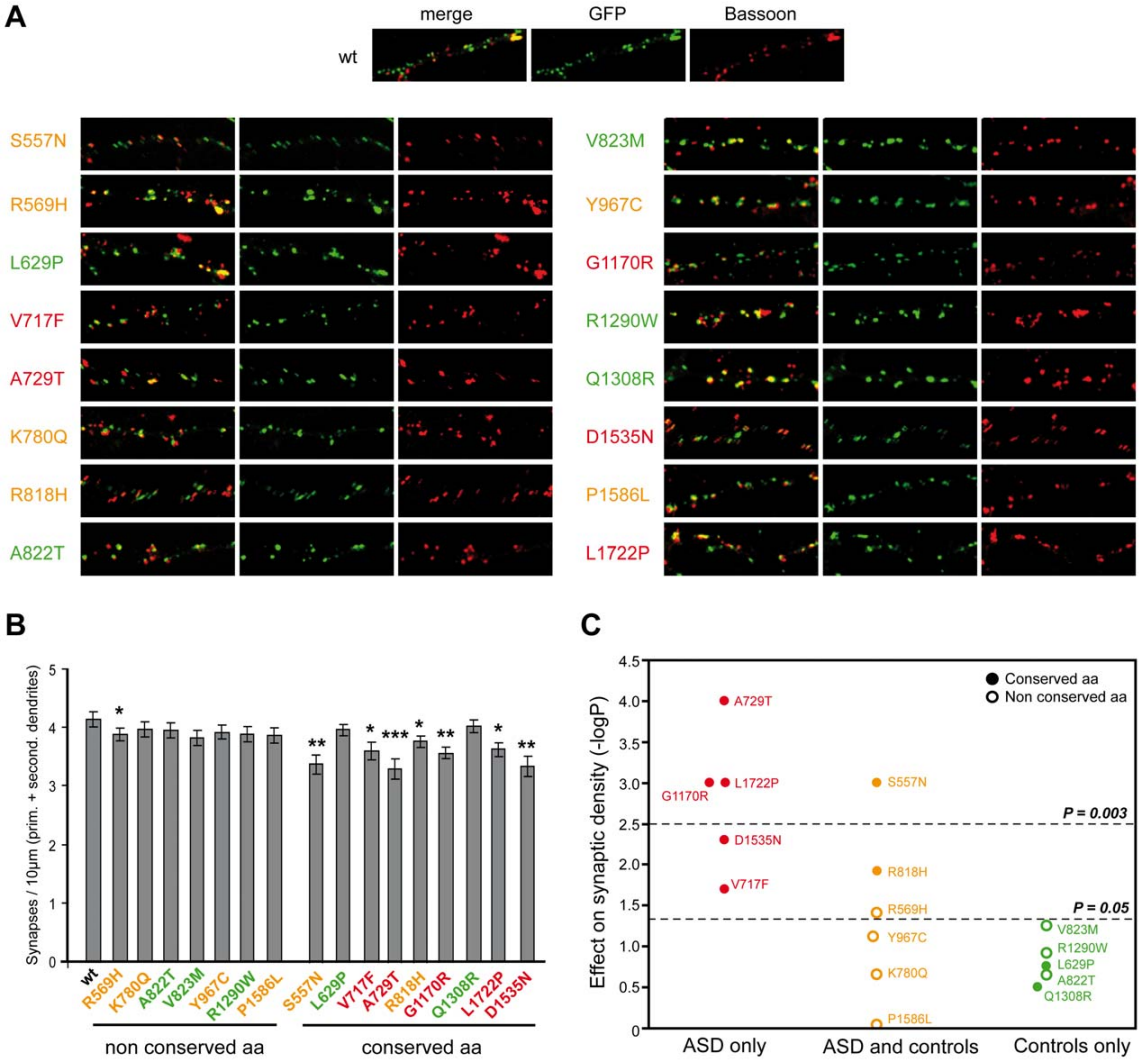
Characterization of the functional impact of *SHANK2* mutations in cultured neuronal cells. A. The colocalization of *ProSAP1A/SHANK2*-EGFP (postsynaptic marker) and Bassoon (presynaptic marker) indicated that the mutations did not disturb the formation of SHANK2 clusters at excitatory synapses along the dendrites. B. The quantification of synapse density was performed on 20 transfected hippocampal neurons per construct from at least three independent experiments. The majority of the *ProSAP1A* variants affecting a conserved amino acid among SHANK proteins reduced significantly the synaptic density compared with the variants that affect amino acid non conserved among SHANK proteins (Mann-Whitney U-test: n_WT_ = 20, n_mut_ = 20; U_S557N_ = 82.5, p_S557N_ = 0.001; U_R569H_ = 124, p_R569H_ = 0.04; U_L629P_ = 149, p_L629P_ = 0.17; U_V717F_ = 114, p_V717F_ = 0.02; U_A729T_ = 73, p_A729T_ = 0.000; U_K780Q_ = 154, p_K780Q_ = 0.221; U_R818H_ = 108, p_R818H_ = 0.012; U_A822T_ = 154.5, p_A822T_ = 0.224; U_V823M_ = 129, p_V823M_ = 0.056; U_Y967C_ = 134, p_Y967C_ = 0.076; U_G1170R_ = 78, p_G1170R_ = 0.001; U_R1290W_ = 142, p_R1290W_ = 0.121; U_Q1308R_ = 162, p_Q1308R_ = 0.314; U_D1535N_ = 97, p_D1535N_ = 0.005; U_P1586L_ = 137, p_P1586L_ = 0.910; U_L1722P_ = 79, p_L1722P_ = 0.001, *p<0.05, **p<0.01, ***p<0.001). **C.** Effect of the variants on synaptic density. The y-axis represents −log P compared to WT (P obtained with Mann-Whitney test). After Bonferroni correction for 16 tests, only P values<0.003 were considered as significant. Variants represented in red were specific to ASD, in orange were shared by ASD and controls, and in green were specific to the controls. Open circles and filled circles represent non conserved and conserved amino acids, respectively. Prim, primary; second, secondary.

In order to establish the functional impact of *SHANK2* variations, we performed expression studies in primary neuronal cell cultures after over-expression of wild-type *vs.* mutant *ProSAP1A/Shank2A* cDNA (Figure 4). All the variants (n = 16) identified by our first screen of 230 patients and 230 controls were tested: 5 were identified only in patients (V717F, A729T, G1170R, D1535N and L1722P), 6 were detected in patients and controls (S557N, R569H, K780Q, R818H, Y967C and P1586L) and 5 were only found in controls (L629P, A822T, V823M, R1290W and Q1308R). All variants were predicted as damaging by Polyphen2 DIV except Q1308R identified only in controls and predicted as benign [41]. In the patient sample, 5/5 variants affected conserved amino acids in the SHANK proteins compared with only 2/6 in the group of variants identified in patients and controls, and 2/5 in the control group. All mutation sites were introduced into the rat *ProSAP1A* cDNA and confirmed by sequencing. The effect of the *Shank2* variants was further investigated in cultured hippocampal neurons. Upon transfection, Western blot analysis revealed that the different GFP-Shank2 fusion proteins were expressed with the expected size (Figure S4). Results from quantification showed that none of the variants affected the cluster formation of Shank2 protein along the dendrites, the number or the general branching pattern of dendrites (Figure S4). In contrast, 8 variants identified in patients or in patients and control group reduced significantly the density of Shank2 positive synapses per 10 µm dendrite length compared with wild-type GFP-Shank2 (Figure 4). None of the variants identified in controls only were shown to have a significant effect. After Bonferonni correction for the 16 tests, 4 variants significantly affected synapse density. Among these variants, A729T, G1170R and L1722P were identified only in patients and S557N was observed more frequently in patients than in controls. As expected, the majority of the variants leading to reduced synaptic density altered conserved amino acids present in SHANK proteins (7/8), and the majority of variants not changing synaptic density affected amino acids present only in SHANK2 (7/8). The 4 strongly associated (after Bonferroni correction) variants affecting synaptic density modified conserved amino acids in other SHANK proteins. Because the significant threshold of 0.05 is arbitrary, we additionally tested for the quantitative effect of the variant on synaptic density as a continuous trait (Figure S4) and found that variants identified in patients were associated with a significant decrease of synapse density *in vitro* compared with those shared by patients and controls (Student's t test 2-sided P = 0.022) or those only detected in the controls (Student's t test 2-sided P = 0.0013). As expected, variants affecting conserved amino acids were associated with a higher reduction of synapse density *in vitro* (Student's t test 2-sided P = 0.014).

### Additional CNVs affect neuronal genes in patients with *de novo SHANK2* deletions and in the control carrying the *SHANK2* splice mutation

**Figure 5:**
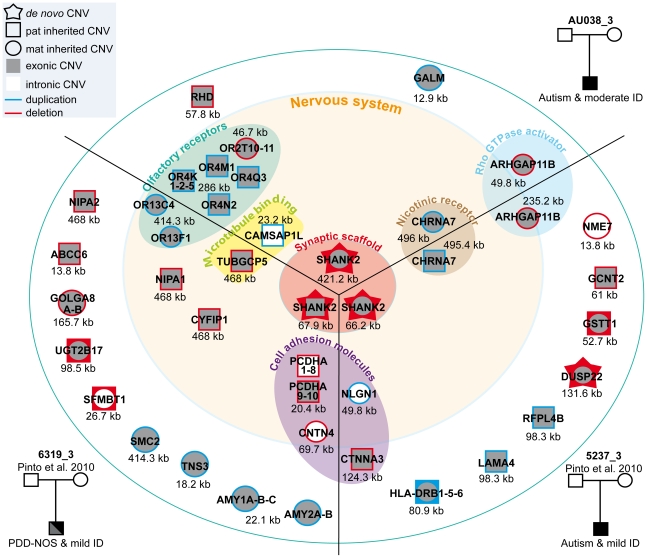
Characterization of CNVs in three patients carrying a *de novo* deletion of *SHANK2*. Paternally or maternally inherited CNVs are indicated by squares and circles, respectively. *De novo* CNVs are indicated by stars. Deletions and duplications are indicated in red and blue, respectively. CNVs hitting exons or only introns are filled with grey and white, respectively. Squares and circles within star represent *de novo* CNV of paternal or maternal origin; circles within squares represent CNV inherited by father or mother. ABCC6, ATP-binding cassette, sub-family C, member 6 pseudogene 2; ADAM, ADAM metallopeptidase; AMY1, amylase (salivary); AMY2A, amylase (pancreatic); ARHGAP11B, Rho GTPase activating protein 11B; CAMSAP1L1, calmodulin regulated spectrin-associated protein 1-like 1; CHRNA7, cholinergic receptor, nicotinic, alpha 7; CNTN4, contactin 4; CTNNA3, catenin (cadherin-associated protein), alpha 3; CYFIP1, cytoplasmic FMR1 interacting protein 1; DUSP22, dual specificity phosphatase 22; GALM, galactose mutarotase; GCNT2, glucosaminyl (N-acetyl) transferase 2; GOLGA, golgi autoantigen, golgin subfamily a; GSTT1, glutathione S-transferase theta 1; HLA-DRB, major histocompatibility complex, class II, DR beta; LAMA4, laminin, alpha 4; NIPA, non imprinted in Prader-Willi/Angelman syndrome; NLGN1, neuroligin 1; NME7, non-metastatic cells 7; OR, olfactory receptor; PCDHA, protocadherin alpha; RFPL4B, ret finger protein-like 4B; RHD, Rh blood group, D antigen; SFMBT1, Scm-like with four mbt domains 1; SHANK2, SH3 and multiple ankyrin repeat domains 2; SMC2, structural maintenance of chromosomes 2; TNS3, tensin 3; TUBGCP5, tubulin, gamma complex associated protein 5; UGT2B17, UDP glucuronosyltransferase 2 family, polypeptide B17.

**Figure 6:**
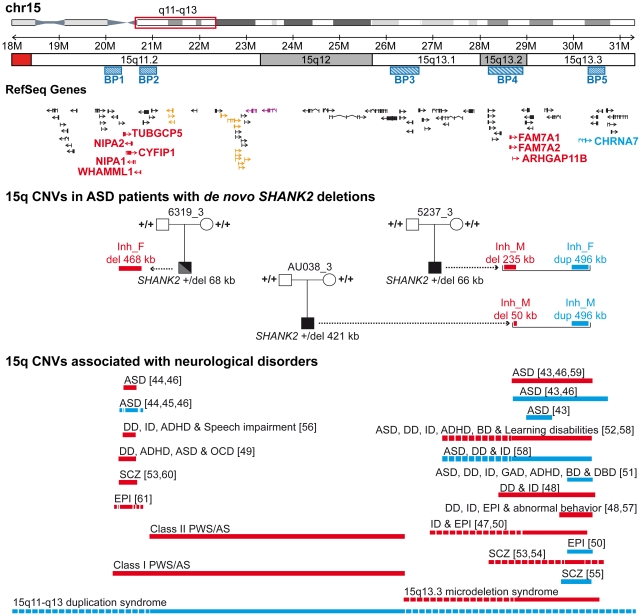
Inherited 15q11–q13 CNVs identified in three ASD patients carrier of a *de novo* SHANK2 deletion. Deletions (del) and duplications (dup) are indicated in red and blue, respectively. Paternally and maternally imprinted genes are indicated in yellow and pink, respectively. Genes altered by the CNVs are indicated in blue or red. The bottom part of the figure indicates the location of the deletions/duplications previously associated with neuropsychiatric disorders [43]–[61]. BP, breakpoint; Inh_M, inherited by mother; Inh_F, inherited by father; AS, Angelman syndrome; ASD, Autism spectrum disorders; ADHD, attention deficit-hyperactivity disorder; BP, bipolar disorder; DD: developmental delay; DBD, disruptive behavior disorder; EPI, epilepsy; GAD, generalized anxiety disorder; OCD, obsessive-compulsive disorder; ID, intellectual disability; PWS, Prader-Willi syndrome; SCZ, schizophrenia.

To test if additional CNVs may modulate the impact of *SHANK2* mutations in the development of ASD, we analyzed the CNVs of patient AU038_3 and the two patients (5237_3 and 6319_3) carrying *SHANK2 de novo* deletions previously identified by Pinto et al. [9] (Figure 5 and Table S6). In addition to our CNV study group of 260 patients with ASD and 290 controls, we used the CNV dataset from the AGP, which includes 996 patients with ASD and 1287 controls genotyped with the Illumina 1M SNP array [9]. Remarkably, all three patients with *SHANK2 de novo* deletions also carried rare inherited genetic imbalances at chromosome 15q11–q13 (Figure 6), a region associated with Angelman syndrome, Prader-Willi syndrome and other neuropsychiatric disorders, including ASD [42]–[61]. This region is characterized by recurrent deletions/duplications with breakpoints generally located within five segmental duplications named BP1 to BP5, which act as hotspots of non-allelic homologous recombination. In the BP5 region, patients AU038_3 and 5237_3 carried the same 496 kb duplication of the nicotinic receptor *CHRNA7* gene (29.8–30.3 Mb, hg 18; maternally inherited in patient AU038_3 and paternally inherited in patient 5237_3). This small *CHRNA7* duplication was present in 13 of 1257 patients with ASD (1.03%) compared with 9 of 1577 controls (0.57%) (Fisher's exact test, 2-sided P = 0.19).

These duplications are considered of uncertain clinical significance since they were previously detected at similar frequencies in patients with epilepsy (6 of 647, 0.93%), in controls (19 of 3699, 0.51%) [50], and in subjects referred for chromosomal microarray analysis (55 of 8832, 0.62%) [51]. In contrast, larger 15q13.3 deletions (∼1.5 Mb) between BP4 and BP5, encompassing the *CHRNA7* locus have been associated with disorders such as ID, epilepsy, schizophrenia, and ASD [43], [46]–[48], [50], [52]–[54], [57]–[59]. In the BP4 region, the same two patients AU038_3 and 5237_3 also carried two independent deletions of the rhoGAP *ARHGAP11B* gene. Loss of *ARHGAP11B* was detected in 8 of 1257 patients with ASD (0.64%) and in 4 of 1577 controls (0.25%) (Fisher's exact test, 2-sided P = 0.15). Patient 5237_3 carried a large deletion (235.2 kb) of the full gene, transmitted by the mother. Patient AU038_3 carried a smaller deletion of 49.8 kb of the first two exons, transmitted by the mother. Both deletions overlap the segmental duplications of BP4 and have been reported to accompany the majority of microduplications involving *CHRNA7* [51]. However, in patient 5237_3, the two CNVs are present on distinct parental chromosomes since the *CHRNA7* duplication and the *ARHGAP11B* deletion are paternally and maternally inherited, respectively. Finally, the third patient, 6319_3, carried a paternally-inherited BP1-BP2 deletion of 468 kb, removing *NIPA1*, *NIPA2*, *CYFIP1*, and *TUBGCP5*. This deletion was observed in 4 of 1257 patients with ASD (0.32%) and in 4 of 1577 controls (0.25%) (Fisher's exact test, 2-sided P = 0.74). The BP1-BP2 deletion is associated with phenotypic variability and has been reported in individuals with neurodevelopmental disorders [20], schizophrenia [53], [60], ASD [44]–[46], [49], and epilepsy [61]. In a recent screen for large CNVs (>400 kb) performed on 15,767 children with ID and various congenital defects, and 8,329 unaffected adult controls [20], deletions affecting *CYFIP1*, *NIPA1*, *NIPA2* and *TUBGCP5* were associated with neurodevelopmental disorder (P = 4.73×10^−6^), epilepsy (P = 1.48×10^−3^) and autism (P = 1.99×10^−2^).

Several additional CNVs also altered compelling candidate genes for susceptibility to ASD. In patient AU038_3 we detected a previously unreported paternally inherited intronic duplication of *CAMSAP1L* on chromosome 1q32.1, coding for a calmodulin regulated spectrin-associated protein highly expressed in the brain. Patient 5237_3 carried a *de novo* deletion altering the coding sequence of the tyrosine phosphatase *DUSP22* on chromosome 6p25.3 and a maternally inherited intronic duplication of *NLGN1* on chromosome 3q26.3 [9]. These CNVs were observed at similar frequencies in patients with ASD compared with controls. *DUSP22* deletions were observed in 8 of 1257 patients with ASD (0.64%) and in 14 of 1577 controls (0.89%), while *NLGN1* intronic duplications were observed in 60 of 1257 patients with ASD (4.77%) and in 62 of 1577 controls (3.93%). Finally, patient 6319_3 carried an unreported maternally inherited intronic deletion of contactin *CNTN4*, a gene on chromosome 3p26.3 associated with ASD [62], as well as a paternally inherited deletion within the protocadherin *PCDHA1-10* gene cluster on chromosome 5q31.3. Interestingly, this deletion removes the first exon of both *PCDH8* and *PCDH9* and was significantly less frequent in patients with ASD compared with controls (ASD: 62 of 1257; controls: 132 of 1577; Fisher's exact test, 2-sided P = 0.0003; OR = 0.57; 95% CI = 0.41–0.78).

We also analyzed the genome of the Swedish control SWE_Q56_508 carrying the *SHANK2* splice mutation using the Human Omni2.5 BeadChip array from Illumina (Figure 3B). Two close duplications on 2p25.3 were detected, altering four genes, *LOC391343*, *SNTG2*, *PXDN* and *MYT1L*. The inheritance of these two duplications could not be investigated, because DNA samples from the parents were not available. However, 2 of 1577 controls also carried of the same close duplications, suggesting that these CNVs are located on the same chromosome. Among the affected genes, syntrophin-γ2 (*SNTG2*) and myelin transcription factor 1-like (*MYT1L*) are expressed in the brain. Alterations of *SNTG2* and *MYT1L* have been previously reported in patients with ASD [20], [63], [64] and schizophrenia [65], respectively. SNTG2 is a scaffolding protein interacting with the NLGN3/4X proteins [66] and a component of the dystrophin glycoprotein complex [67]. MYT1L is a myelin transcription factor required to convert mouse embryonic and postnatal fibroblasts into functional neurons [68].

## Discussion

### Deleterious *SHANK2* variations are enriched in patients with ASD, but also observed in controls

The identification of mutations in synaptic proteins such as NRXN1, NLGN3/4X and SHANK2/3 has demonstrated that a synaptic defect might be at the origin of ASD [5], [6]. Here we confirm the presence of *SHANK2 de novo* deletions in individuals with ASD, with a prevalence of 0.38% (1/260) in our cohort of ASD patients analyzed with the Illumina 1M SNP array. This frequency is similar to the one reported previously by the AGP in a larger sample of 996 patients with ASD (0.2%) [9]. *SHANK2* deletions altering exons were not detected in controls, in agreement with previous findings [9], [26]. As reported for *SHANK3* [7], no other coding variations were detected in the remaining *SHANK2* allele of the deletion carriers, suggesting that, in some individuals, a *de novo* deletion of a single allele of *SHANK2* might be sufficient to increase the risk for ASD. In one case, a patient carried two rare *SHANK2* variants predicted as deleterious and inherited from different parents, indicating that they were separate alleles.

For the remaining *SHANK2* variants, patients were heterozygous for non-synonymous rare variations inherited from one of their parents (Figure S3). Since parents were apparently asymptomatic, the causative role of these variants in ASD remains difficult to ascertain. However, we observed a significant enrichment of *SHANK2* variants affecting conserved amino acids in patients with ASD compared with controls. This was also the case in the previous mutation screening by Berkel *et al.* 2010 [26]. The majority of the variants affecting conserved residues and identified in the patients were shown to alter the ability of *SHANK2* to increase the number of synapses *in vitro*. Importantly, the assays performed in this study show that the variants can potentially impact on the function of the protein, but they do not confirm that they have deleterious effects on neuronal function *in vivo* in people that carry them. However, these results are consistent with previous findings showing that inherited variants of SHANK2 and SHANK3 cause synaptic defects *in vitro* [7], [69], [70]. Recently, Berkel *et al.* 2011 showed that two inherited (L1008_P1009dup, T1127M) and one *de novo* (R462X) SHANK2 mutations identified in patients with ASD affect spine volume and reduced Shank2 cluster sizes [70]. This deleterious effect was also observed *in vivo* since mice expressing rAAV-transduced Shank2-R462X present a specific long-lasting reduction in miniature postsynaptic AMPA receptor currents [70].

In patients, the only feature associated with carriers of *SHANK2* mutations compared with other patients was a trend for low IQ (P = 0.025, OR = 3.75, 95% CI = 1.1–20.0) (Table S8). But, as observed for *SHANK3* mutations, this correlation could differ from one individual to another (i.e. the patient with a *SHANK2 de novo* stop mutation reported by Berkel *et al.* 2010 presented with high-functioning autism [26]).

Our result also showed that potentially deleterious *SHANK2* variants were detected in a heterozygous state in parents and in the general population without causing severe phenotypic consequences. Indeed, we showed that almost 5% of the Finnish population is heterozygous for the *SHANK2* R818H variation, which modifies a conserved amino acid and is associated with lower synaptic density *in vitro*. Furthermore, we identified a *SHANK2* splice site mutation in a control female without any apparent psychiatric disorders. Similarly, two frame-shift mutations and one splice site mutation of *SHANK2* are listed in dbSNP and in the 1000 genomes project [71]. These nonsense variations should be interpreted with caution since none of them has been validated by Sanger sequence technology. Taken together, variants affecting conserved amino acids of SHANK2 might act as susceptibility variants for ASD, but, in some cases, additional genetic, epigenetic or environmental factors seem to be necessary for the emergence of the disorder.

### Additional CNVs in subjects with *SHANK2* mutations may modulate the risk for ASD

In order to detect risk and protective genetic factors, we analyzed the CNV burden of the individuals carrying deleterious variations of *SHANK2*. Notably, the three ASD patients with *de novo SHANK2* deletions also carried CNVs on chromosome 15q11–q13, a region associated with ASD [43], [47], [48], [50]–[52], [72]. In contrast, the patient reported by Berkel *et al.* 2010, who did not meet all the diagnostic criteria for ASD, seemed to have no CNV at chromosome 15q [26]. Although the probability to observe the co-occurrence of a *de novo SHANK2* deletion and a duplication of *CHRNA7* at 15q is very low, two of the three patients carrying a *de novo SHANK2* deletion also carried the *CHRNA7* duplication. While the numbers are small, this finding could suggest epistasis between these two loci. The role of *CHRNA7* in ASD was recently supported by the observation of low levels of *CHRNA7* mRNA in the post-mortem brain from patients with ASD [73]. Interestingly, it was also found that, in contrast to the gene copy number, the transcript levels *CHRNA7* were reduced in neuronal cells [74] or brain samples with maternal 15q duplication [75]. Finally, functional studies have shown that NLGN and NRXN, which belong to the same synaptic pathway, are key organizers of the clustering of nicotinic receptors at the synapse [76]–[78]. Therefore the co-occurrence of a deletion of *SHANK2* and a duplication of the nicotinic receptor *CHRNA7* could act together within the same pathway to increase the risk of ASD in patients AU038_3 and 5237_3. In patient 6319_3 carrying the BP1–BP2 deletion, several genes might also play a role in the susceptibility to ASD. Among them, *NIPA1* and *TUBGCP5* encoding a magnesium transporter and a tubulin gamma associated protein, respectively, are highly expressed in the brain. However, the most compelling candidate in the deleted region is *CYFIP1* [45], [53], which codes for a binding partner of FMRP, the protein responsible for fragile X syndrome. Both CYFIP1 and FMRP are involved in the repression of synaptic translation [79], one of the major biological mechanisms associated with ASD [80]. Therefore, the co-occurrence of a loss of one copy of *SHANK2* and *CYFIP1* might increase the risk of abnormal synaptic function in patient 6319_3.

If some individuals have a higher risk to develop ASD when a deleterious *SHANK2* variant is present, others individuals may experience a protective effect by additional genetic factors. For example, control SWE_Q56_508 carried a *SHANK2* splice mutation, but clinical examination revealed no major disorders. In addition, this control individual also carried a partial duplication of *SNTG2* and *MYT1L*. Based on a single control subject, it is not possible to formally prove that these additional hits at *SNTG2* and/or *MYT1L* acted as suppressor mutations, counteracting the phenotypic effects of the *SHANK2* splice mutation. However, the encoded proteins may interact with the NRXN-NLGN-SHANK pathway. Both SNTG2 and SHANK2 are scaffolding proteins localized in actin rich structures [81]–[83] and bind directly to neuroligins [66]. Furthermore, mutations of *NLGN3/4X* identified in patients with ASD decrease their protein binding to SNTG2 [66]. In addition, MYT1L is a myelin transcription factor that is sufficient, with only two other transcription factors, ASCL1 and BRN2, to convert mouse embryonic and postnatal fibroblasts into functional neurons *in vitro* [67]. Therefore, alterations of SNTG2 and/or MYTL1 might modulate synapse physiology and counteract the effect of the *SHANK2* splice site mutation. We recently highlighted the key role of synaptic gene dosage in ASD and the possibility that a protein imbalance at the synapse could alter synaptic homeostasis [6]. In the future, animal models should be developed to test whether the effect of a primary mutation in a synaptic protein complex (e.g. *Shank2*) can be reduced or suppressed by a second mutation (e.g. *Sntg2* or *Myt1l*). A similar suppressor effect has been demonstrated by the decrease of abnormal behavior of the *Fmr1* mutant mice carrying a heterozygous mutation of the metabotropic glutamate receptor mGluR5 [84].

### Conclusions and perspectives

In summary, we confirmed that *de novo SHANK2* deletions are present in patients with ASD and showed that several *SHANK2* variants reduce the number of synapses *in vitro*. The genomic profile of the patients carrying deleterious *de novo SHANK2* deletions also points to a possible genetic epistasis between the NRXN-NLGN-SHANK pathway and 15q11–q13 CNVs. *CHRNA7* and *CYFIP1* were already proposed as susceptibility genes for neuropsychiatric disorders [43], [45], [49], [51], and our study provides additional support for this association. Therefore, as previously observed for ID [85], our results suggest that the co-occurrence of *de novo* mutations, together with inherited variations might play a role in the genetic susceptibility to ASD. Finally, our analyses suggest the interesting possibility that deleterious mutations of neuronal genes (e.g. *SNTG2* and *MYT1L*) could potentially counteract the effect of synaptic deleterious mutations (e.g. *SHANK2*). The identification of risk and protective alleles within the same subject is one of the main challenges for understanding the inheritance of ASD. Initial results from the 1000 genomes project has estimated that, on average, each person carries approximately 250 to 300 loss-of-function variants in annotated genes and 50 to 100 variants previously implicated in inherited disorders [71]. To date, it is not clear how many loci can regulate synaptic homeostasis and how these variants interact with each other to modulate the risk for ASD [6]. A better knowledge of these genetic interactions will be necessary to understand the complex inheritance pattern of ASD.

## Materials and Methods

### Ethics statement

This study was approved by the local Institutional Review Board (IRB) and written inform consents were obtained from all participants of the study. The local IRB are the “Comité de Protection des Personnes” (Île-de-France Hôpital Pitié-Salpêtrière Paris) for France; the Sahlgrenska Academy Ethics committee, University of Gothenburg for Sweden; the local IRB of the medical faculty of JW Goethe University Frankfurt/Main for Germany; the Committee #3 of the Helsinki University Hospital, Finland; the “Comitato Etico IRCCS Fondazione Stella Maris” at Stella Maris Institute, Calambrone (Pisa), Italy; the “Comitato Etico Azienda Ospedaliera-Universitaria Policlinico-Vittorio Emanuele”, Catania, Italy.

### Patients

Patients with ASD and analyzed for CNV analysis and/or mutation screening are presented in Table S1. Patients were recruited by the PARIS (Paris Autism Research International Sibpair) study at specialized clinical centers disposed in France, Sweden, Germany, Finland, UK. The Autism Diagnostic Interview-Revised (ADI-R) and Autism Diagnostic Observation Schedule (ADOS) were used for clinical evaluation and diagnosis. In Sweden, in some cases, the Diagnostic Interview for Social and Communication Disorders (DISCO-10) was applied instead of the ADI-R. Patients were included after a clinical and medical check-up with psychiatric and neuropsychological examination, standard karyotyping, fragile-X testing and brain imaging and EEG whenever possible. All patients were from Caucasian ancestry.

The patient AU038_3 with a *de novo SHANK2* deletion is an 11.05 year-old boy diagnosed with autism and moderate ID (Table S2). He was the only child of non-consanguineous parents from European descent. His parents had no relevant personal and familial history of psychiatric or medical illness. He was born at 40 weeks of gestation, after normal pregnancy and delivery. Birth weight, length and occipitofrontal head circumference were 2500 g (5th percentile), 48 cm (22nd percentile) and 31 cm (2nd percentile), respectively. Apgar scores were 7 and 10 at 1 and 5 minutes, respectively. In the first year of life, the pediatrician reports did not mention signs of hypotonia. At 2 months, he was operated for an inguinal hernia. Motor acquisition was apparently normal (sitting at 6 months), but with a late acquisition of walking, at 18 months. Speech was severely delayed, without any apparent regressive phase. Only a few words and sentences appeared when he was 4 y and 6.5 y, respectively. His expressive language remained limited to restrictive sentences, mainly dyssyntaxic. A formal diagnostic assessment for autism was performed when he was 11 years old. The scores of the Autism Diagnostic Interview-Revised (ADI-R) domains were: social 24, communication 23, and behavior 6 (cut-offs for autism diagnosis are 10, 8 (verbal autism) and 3, respectively); the age at first symptoms was before 36 months. Cognitive evaluation with the Kaufman Assessment Battery for Children (K-ABC) showed moderate intellectual deficit (composite score 40). He required assistance with basic activities such as eating and dressing. At examination, he had a normal facial appearance, with a prominent chin. General and neurological examinations were normal, except for hypermetropia and astigmatism. High-resolution karyotype, fragile X testing, MLPA analysis of telomeres and microdeletion/microduplication syndromes, and metabolic screening for inherited disorders of metabolism (urine amino acids, mucopolysaccharides and organic acids, uric acid in blood and urine) were all normal. No significant epileptic event was reported on the electroencephalogram.

The two male patients with *de novo SHANK2* deletions reported by Pinto *et al.* 2010 [9] (5237_3 and 6319_3) shared several clinical features with patient AU038_3. Patient 5237_3 is a Canadian subject diagnosed with autism (based on ADI-R and ADOS) associated with below average non verbal IQ (<1^st^ percentile) and language (<1^st^ percentile). He had minor dysmorphic features including 5^th^ finger clinodactyly and several curled toes, and no history of epilepsy. Patient 6319_3 was recruited in the same geographic area as patient AU038_3 (Grenoble, France) and was clinically diagnosed with PDD-NOS. The ADI-R scores were: social 14, communication 8, behaviors 2 (cut-off for autism: 3); with an age at first symptoms <36 months). He had mild ID as evaluated with the WISC-III (full scale IQ 60, performance IQ 60, verbal IQ 67). His language was delayed (first words 24 m, first sentences 48 m), but functional. He had no history of regression or epilepsy. The physical exam was normal, except for large and prominent ears and flat feet; the neurological exam was also normal. Similarly to patient AU038_3, he had hypermetropia.

The control female carrying the splice site mutation (IVS22+1G>T) was part of a cohort of 172 females recruited for a study on obesity, anthropometrics, and cardiovascular risk factors [37]. In addition, these women were assessed for axis I psychiatric disorders and for personality traits using the Temperament and Character Inventory (TCI) [38] and the Karolinska Scales of Personality (KSP) [39]. This subject had no psychiatric disorders and her TCI and KSP scores were similar to those found in the general population.

### Genomic structure and transcripts analysis of *SHANK2*

To define the genomic structure of the human *SHANK2* gene, we used the two reference sequence genes from UCSC (*NM_012309* and *NM_133266*), one human mRNA from GenBank (*DQ152234*) and three Rattus reference sequence genes from UCSC (*NM_201350*, *NM_133441* and *NM_133440*). *SHANK2* is transcribed in four isoforms described in GenBank (*AB208025*, *AB208026*, *AB208027* and *AF141901*) and is composed of 25 exons. Transcript analysis of *SHANK2* was performed in human brain regions from four independent controls (two females and two males) and in human tissues (heart, brain, placenta, lung, liver, skeletal muscle, kidney, pancreas and B lymphoblastoid cell lines) using the Clontech Multiple Tissue cDNA panel (Clontech). Total RNA was isolated from control human brain tissues by the acid guanidinium thiocyanate phenol chloroform method and reverse transcribed by oligodT priming using SuperScript II Reverse Transcriptase (Invitrogen). The PCR was performed with HotStar Taq polymerase (Qiagen) and the protocol used was 95°C for 15 min, followed by 40 cycles at 95°C for 30 sec, 55 to 58°C for 30 sec, 72°C for 30 sec to 1 min, with a final cycle at 72°C for 10 min. PCR primers were designed to detect the ANK domain, the SH3 domain, the PDZ domain, and the SAM domain in order to distinguish the four *SHANK2* isoforms and are indicated in Table S11. All RT-PCR products were directly sequenced. The expression of *SHANK2E* isoform was also studied by SYBR-Green real-time PCR approach. The fluorescence was read with the Applied Biosystems 7500 Real-Time PCR System. Each assay was conducted in three replicates. GAPDH was used for the ΔCt calculation and total brain was used as the reference for relative quantification calculation (RQ). The relative RQ of transcripts was calculated as 2^−ΔΔCT^ with the magnitude of upper error as 2^−(ΔΔCT−SEM)^-2^−ΔΔCT^ and the magnitude of lower error as 2^−ΔΔCT^-2^−(ΔΔCT+SEM)^. The primers specific to *SHANK2E* isoform are indicated in Table S11. *In situ* hybridization was performed essentially as described previously [28]. Transcripts encoding the different *ProSAP1/Shank2* cDNAs (*ProSAP1/Shank2* starting with the PDZ domain, *ProSAP1A*, starting with the SH3 domain and *ProSAP1E/Shank2E*, starting with the ankyrin repeats) were detected with isoform specific S35 labeled cDNA antisense oligonucleotides purchased from MWG-Biotech (Ebersberg, Germany) directed against the ATG regions of the different mRNAs. All variants were evaluated for potential pathogenicity using the HumDIV method for rare alleles of PolyPhen2 [41].

### CNV detection and validation

DNA was extracted from blood leukocytes or B lymphoblastoid cell lines. The *SHANK2* CNV was detected with the Illumina Human 1M-Duo BeadChip, which interrogates 1 million SNPs distributed over the human genome. For the Swedish control SWE_Q56_508 carrying the *SHANK2* splice mutation we used the Illumina Human Omni2.5 BeadChip array. The genotyping was performed at the Centre National de Génotypage (CNG) and the Institut Pasteur. Only samples that met stringent quality control (QC) criteria were included: call rate ≥99%; high confidence score log Bayes factor ≥15; standard deviation of the log R ratio (LRR) ≤0.35 and of the B allele frequency (BAF)≤0.13; number of consecutive probes for CNV detection ≥5; CNV size ≥1 kb. When the QC criteria were met, we used two CNV calling algorithms, QuantiSNP [86] and PennCNV [87], and the CNV viewer, SnipPeep (http://snippeep.sourceforge.net/). To obtain high-confidence calls, the CNVs identified by QuantiSNP were validated by visual inspection of the LRR and BAF values. PennCNV was used to confirm inheritance status of the resulting CNV calls. CNVs were validated by quantitative PCR analysis using the Universal Probe Library (UPL) system from Roche. UPL probes were labeled with FAM and the fluorescence was read with the Applied Biosystems 7500 Real-Time PCR System. Each assay was conducted in four replicates for target region probe-set and control region probe-set. Relative levels of region dosage were determined using the comparative CT method assuming that there were two copies of DNA in the control region. The relative copy number for each target region was calculated as 2^−ΔΔCT^ with the magnitude of upper error as 2^−(ΔΔCT−SEM)^-2^−ΔΔCT^ and the magnitude of lower error as 2^−ΔΔCT^-2^−(ΔΔCT+SEM)^. UPL probes and primers are indicated in Table S12. For comparisons between patients and controls, statistical significance for each CNV was assessed using a 2-sided Fisher's exact test.

### Mutation screening

The 24 coding exons of *SHANK2* were amplified and sequenced for mutation screening. The PCR was performed on 20–40 ng of genomic DNA template with HotStar Taq polymerase from Qiagen for all exons the protocol used was 95°C for 15 min, followed by 35–40 cycles at 95–97°C for 30 sec, 55–62°C for 30 sec, 72°C for 30 sec to 90 sec, with a final cycle at 72°C for 10 min. Sequence analysis was performed by direct sequencing of the PCR products using a 373A automated DNA sequencer (Applied Biosystems). Genotyping of R185Q, V717F, A729T, R818H, G1170R, D1535N and L1722P was performed by direct sequencing or Taqman SNP Genotyping Assays system from Applied Biosystems designed with Custom TaqMan Assay Design Tool. All primers are indicated in Table S9. Enrichment of *SHANK2* variations in the ASD sample compared with controls was assessed using a 1-sided Fisher's exact test (hypothesizing that cases will show an excess of *SHANK2* variants compared to controls).

### In vitro mutagenesis and transfection studies in hippocampal neurons

Rat *GFP-ProSAP1A* (*Shank2A*) cDNA was mutated according to the human mutations using the site directed mutagenesis kit (Stratagene). The mutagenesis primers were listed in Table S10. We have tested all the variants (n = 16) identified in our first screen of 230 patients with ASD and 230 controls: 5 were detected only in patients (V717F, A729T, G1170R, D1535N and L1722P), 6 were detected in patients and controls (S557N, R569H, K780Q, R818H, Y967C and P1586L) and 5 were only found in controls (L629P, A822T, V823M, R1290W and Q1308R). All mutated amino acids were conserved among human, rat and mouse ProSAP1/Shank2. All cDNAs were sequenced and subsequently tested for expression by Western blot analysis. After expression of the constructs in Cos7 cells, the cell homogenate was separated on a gel, transferred to a nitrocellulose membrane and subsequently protein bands were detected using a rabbit anti-GFP antibody. Thereafter, the cDNAs were transfected into primary hippocampal neurons. Cell culture experiments of rat hippocampal primary neurons (embryonic day 18–21: E18-21) were performed as described previously [88]. In brief, after preparation, hippocampal neurons were seeded on poly-l-lysine (0.1 mg/ml; Sigma-Aldrich, Steinheim, Germany) coated coverslips at a density of 4×10^4^ cells/well (transfection experiments) or 2×10^4^ cells/well (immunological staining). Cells were grown in Neurobasal medium (Invitrogen, Karlsruhe, Germany), complemented with B27 supplement (Invitrogen), 0.5 mM L-glutamine (Invitrogen), and 100 U/ml penicillin/streptomycin (Invitrogen) and maintained at 37°C in 5% CO_2_. Hippocampal cells were transfected using Lipofectamine 2000, according to the manufacturer's recommendation (Invitrogen). Fluorescence images were obtained using a camera attached to a fluorescence microscope. For immunofluorescence, the primary cultures were fixed with ice cold 4% paraformaldehyde/1.5% sucrose/PBS for 20 min at 4°C and processed for immunohistochemistry. After washing three times with 1× PBS for 5 min at room temperature the cells were permeabilized for 3 min on ice in a buffer containing 0.1% Triton X-100/0.1% Na-Citrate/PBS and washed again three times with 1× PBS. Blocking was performed with 10% fetal calf serum/PBS for 1 h at room temperature followed by incubation with the primary antibody (mouse anti-Bassoon) overnight at room temperature. After a further washing-step the cells were incubated with the secondary antibody coupled to Alexa555 (red) (Molecular Probes, Invitrogen) for 90 min at room temperature, washed first with 1×PBS and then with aqua bidest for 5 min and mounted in Mowiol (with or without DAPI for staining of the nucleus). All animal experiments were performed in compliance with the guidelines for the welfare of experimental animals issued by the Federal Government of Germany, the National Institutes of Health and the Max Planck Society.

### Image acquisition and quantification

In morphological studies, dendrites were considered primary when processes extended directly from the cell body, and secondary when processes branched off primary dendrites. Twenty transfected neurons were chosen randomly for quantification from at least three independent experiments for each construct. Morphometric measurements were performed using Axiovision Zeiss microscope and Axiovision software with a 40× magnification. For the quantification of excitatory synapse number, cells were counterstained with anti-Bassoon antibodies. From randomly chosen transfected neurons, Bassoon-positive spots from primary dendrites were counted and the length of dendrites was measured. The total number of spines was expressed as density per 10 µm length of dendrite. Measured data were exported to Excel software (Microsoft), and the data of each variant were compared by using the Mann-Whitney U test. The comparisons of synaptic density for each phenotypic or conservation categories were performed using the Student's t test.

## Supporting Information

Figure S1 *SHANK2E* expression in human multiple tissue panel and in rat embryos. A. Quantitative RT-PCR in human tissues. Primers and probe were designed to detect *SHANK2E* isoform. GAPDH was used for the ΔCt calculation and total brain was used as the reference for relative quantification calculation (RQ ± SEM). B. *In situ* hybridization of rat fetus sagittal sections with *ProSAP1/Shank2* isoform specific oligonucleotides. The *ProSAP1/Shank2* isoform starting with the PDZ domain is solely expressed in brain, brain stem and medulla. The same holds true for the *ProSAP1A/Shank1A* isoform that starts with the SH3 domain. In some sections bone tissue also gave some moderately positive signals. The expression of *ProSAP1E/Shank1E* (with the ankyrin repeats) is especially seen in the liver and some glandular tissue. In the brain, the *ProSAP1E/Shank2E* mRNA is only detectable within the cerebellum (arrow).
(TIF)

Figure S2 Characterization and validation of the *SHANK2* CNV in family AU038. A. Pedigree of the AU038 family showing that the deletion is *de novo* on the maternal chromosome. SNPs were genotyped using the Illumina 1M duo array. B. *SHANK2* CNV validation by quantitative PCR of exon E4–E6, E15–E17 of *SHANK2* using genomic DNA from the father, mother and the proband of family AU038. Results from QPCR analysis confirmed that the deletion is *de novo* and removes exon E5 to Exon E16. Bars represent mean of RQ ± SEM.
(TIF)

Figure S3 Pedigree of families with ASD carrier of non-synonymous *SHANK2* mutations. Variations specific to ASD or shared by ASD and controls are indicated in red and orange, respectively. Clinical phenotypes are specified in the figure.
(TIF)

Figure S4 Functional analysis of *shank2* mutations in cultured hippocampal neurons. A. Rat hippocampal neurons transfected with wild-type or mutated *ProSAP1A/SHANK2*-EGFP cDNA constructs were detected by EGFP expression. B. The quantification of dendrite number was performed on 20 transfected hippocampal neurons per construct from at least 3 independent experiments. No mutation affected the number of dendrites per neuron (Bars represent mean ± SD, Mann-Whitney U test and Kolmogorov-Smirnov Z test; no significant differences; n_WT_ = 20, n_mut_ = 20). Western blot analysis of *ProSAP1A/SHANK2-EGFP* cDNA constructs revealed similar sizes of WT fusion *ProSAP1A/SHANK2*-EGPF protein and the constructs carrying the mutations. C. The variants identified in patients with ASD were associated with reduced synaptic density compared with those identified in controls. D. The variants affecting conserved amino acids in other SHANK proteins were associated with reduced synaptic density compared with those affecting non conserved amino acids. Variations represented in red were specific to ASD, those in orange were shared by ASD and controls, and those in green were specific to controls.
(TIF)

Table S1 Description of the population of patients with ASD analyzed in this study.
(DOC)

Table S2 Clinical description of the patients carrying predicted deleterious *SHANK2* variations. Abbreviations: ADI-R, Autism Diagnosis Interview-Revised; ASD, autism spectrum disorder; CT, computed tomography; ERG, electroretinogram; F, female; FSIQ, full scale IQ; HC, head circumference; K-ABC, Kaufman Assessment Battery for Children; M, male; MRI, magnetic resonance imaging; MZ, monozygotic; NA, not available; ND, not done; PDD-NOS, pervasive developmental disorder not otherwise specified; PET, positron emission tomography; PIQ, performance IQ; PEP-R, Psychoeducational Profile-Revised; VIQ, verbal IQ; WISC-III, Wechsler Intelligence Scale for Children-Third Edition; WPPSI, Wechsler Preschool and Primary Scale of Intelligence; IQ, intellectual quotient.
(DOC)

Table S3 Frequency of *SHANK2* R818H variation in 3250 patients with ASD and 2013 controls. OR, odds ratio; P, p-value.
(DOC)

Table S4 Frequency of *SHANK2* R818H variation in 948 individuals from the Human Genome Diversity Panel.
(DOC)

Table S5 Evolutionary conservation of SHANK2 protein sequence. Variations identified only in patients with ASD, only in controls or shared by patients and controls are indicated in red, green and orange, respectively. Hu, human; Ch, chimpanzee; Ma, macaque; Ra, rat; Mo, mouse; Ck, chicken; Zn, zebrafinsh; Zf, zebrafish; Xe, xenopus.
(DOC)

Table S6 List of all CNVs observed in ASD patients carrying a *de novo* deletion of *SHANK2*. The 6319_3 and 5237_3 patients were described by the AGP [9]. QSNP, QuantiSNP; PCNV, PennCNV; IP, iPattern.
(DOC)

Table S7 Distribution of *SHANK2* variants affecting conserved or non conserved amino acids. All variants came from this study (26) and from Berkel *et al.* 2010 (24). *Several variants shared by patients with ASD and controls were identified in both studies.
(DOC)

Table S8 Clinical comparison of patients with ASD carrying SHANK2 variants with the rest of the cohort of patients. We used the Wilcoxon test for the continuous traits and the Fisher's exact test (2-sided) for the discontinuous traits. OR is given with 95% confidence interval. OR, odds ratio; P, p-value; ADI-R, Autism Diagnosis Interview revised.
(DOC)

Table S9 Primers used for mutation screening.
(DOC)

Table S10 Primers used for *in vitro* mutagenesis.
(DOC)

Table S11 Primers used for mRNA analysis of *SHANK2* isoforms. * Primers were used for relative quantification study of *SHANK2E* isoform. The other primers were used for RT-PCR analysis of each *SHANK2* isoform.
(DOC)

Table S12 Primers used for CNV validation.
(DOC)
