# Supplementary figures and images for: Genetic and Functional Analyses of SHANK2 Mutations Suggest a Multiple Hit Model of Autism Spectrum Disorders
Source: PLoS Genet. 2012 Feb 9;8(2):e1002521. doi: 10.1371/journal.pgen.1002521 (PMC3276563; doi:10.1371/journal.pgen.1002521)

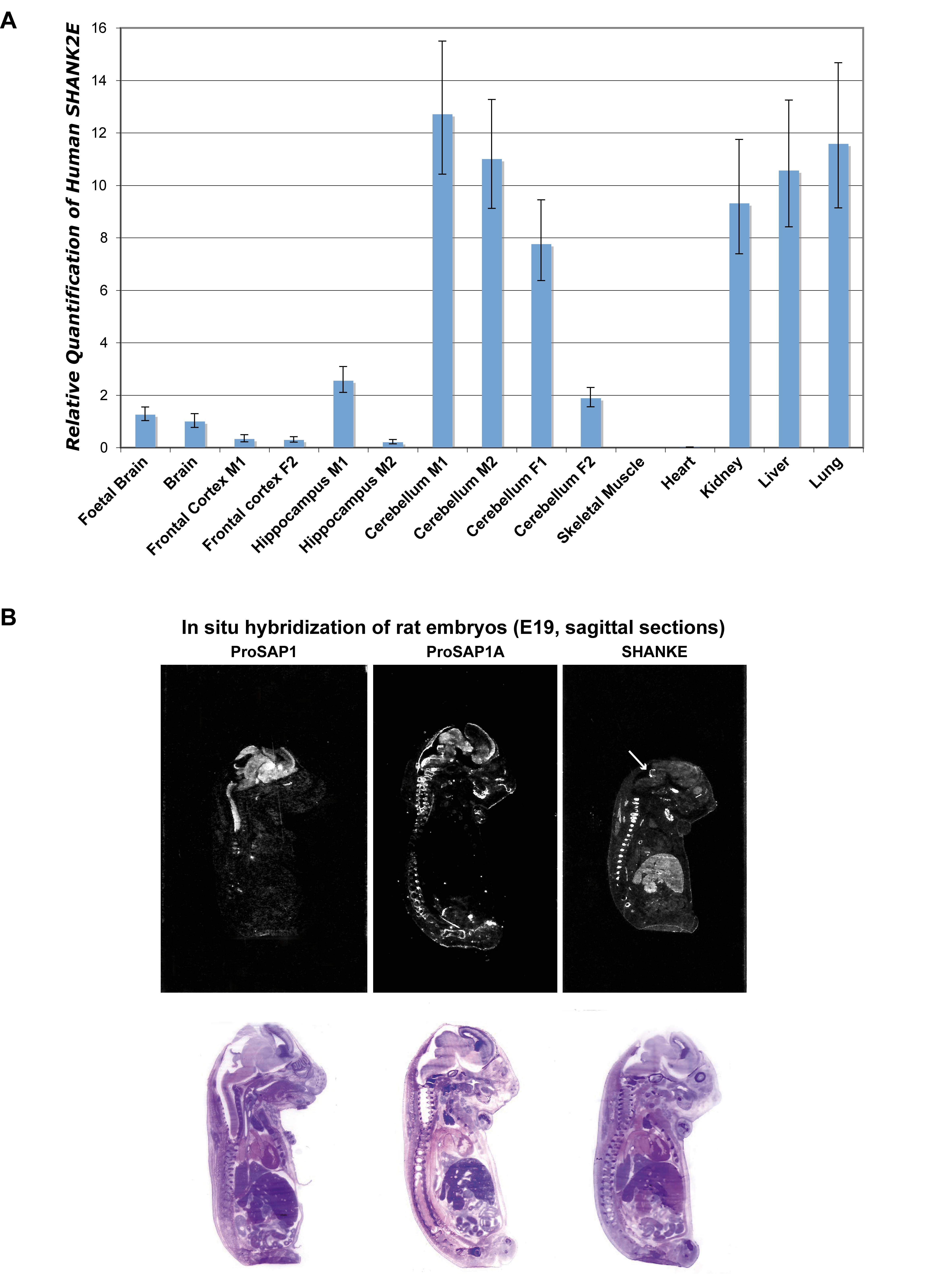

Supplement: Figure S1 — SHANK2E expression in human multiple tissue panel and in rat embryos. A. Quantitative RT-PCR in human tissues. Primers and probe were designed to detect SHANK2E isoform. GAPDH was used for the ΔCt calculation and total brain was used as the reference for relative quantification calculation (RQ ± SEM). B. In situ hybridization of rat fetus sagittal sections with ProSAP1/Shank2 isoform specific oligonucleotides. The ProSAP1/Shank2 isoform starting with the PDZ domain is solely expressed in brain, brain stem and medulla. The same holds true for the ProSAP1A/Shank1A isoform that starts with the SH3 domain. In some sections bone tissue also gave some moderately positive signals. The expression of ProSAP1E/Shank1E (with the ankyrin repeats) is especially seen in the liver and some glandular tissue. In the brain, the ProSAP1E/Shank2E mRNA is only detectable within the cerebellum (arrow). (TIF) [file pgen.1002521.s001.tif]

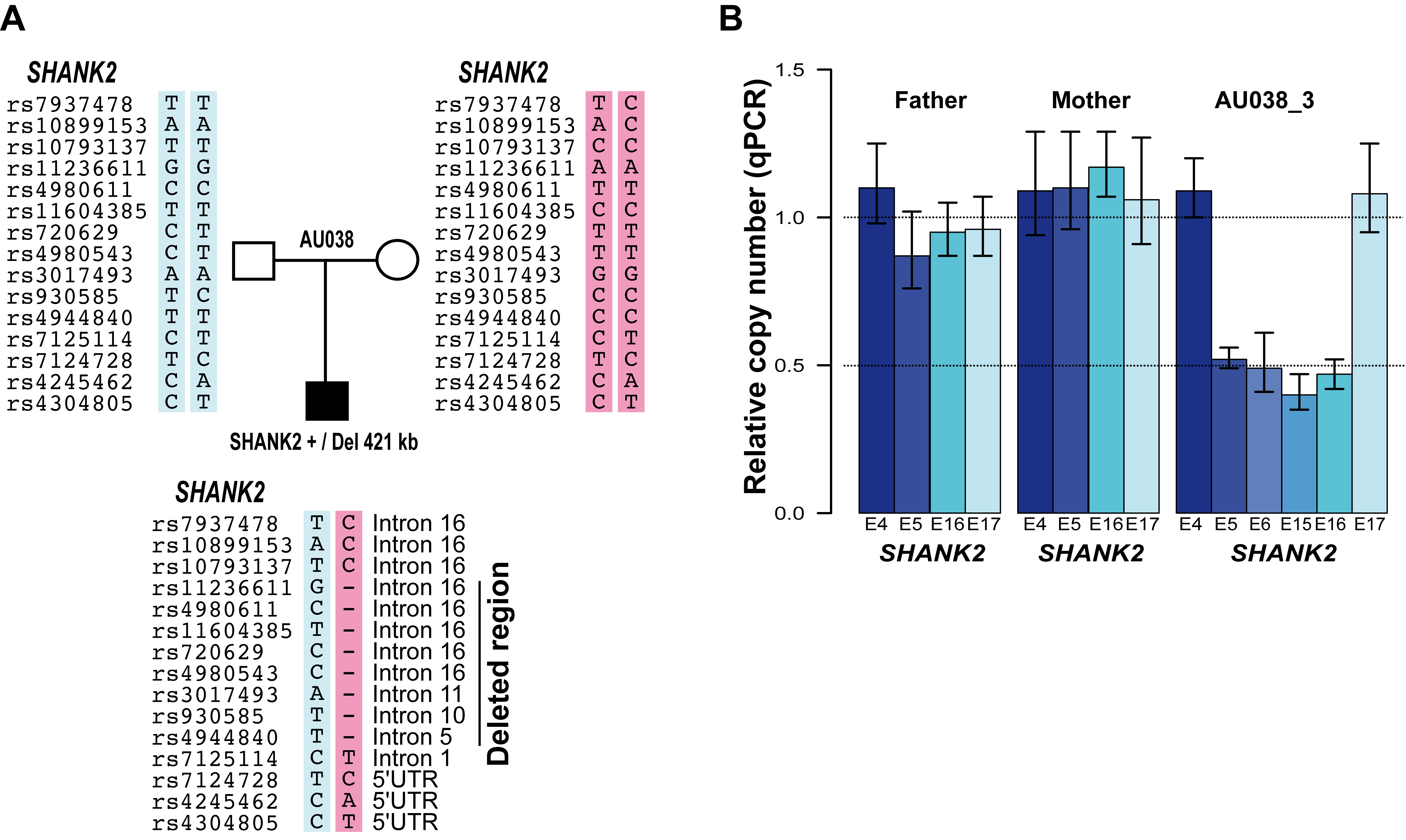

Supplement: Figure S2 — Characterization and validation of the SHANK2 CNV in family AU038. A. Pedigree of the AU038 family showing that the deletion is de novo on the maternal chromosome. SNPs were genotyped using the Illumina 1M duo array. B. SHANK2 CNV validation by quantitative PCR of exon E4–E6, E15–E17 of SHANK2 using genomic DNA from the father, mother and the proband of family AU038. Results from QPCR analysis confirmed that the deletion is de novo and removes exon E5 to Exon E16. Bars represent mean of RQ ± SEM. (TIF) [file pgen.1002521.s002.tif]

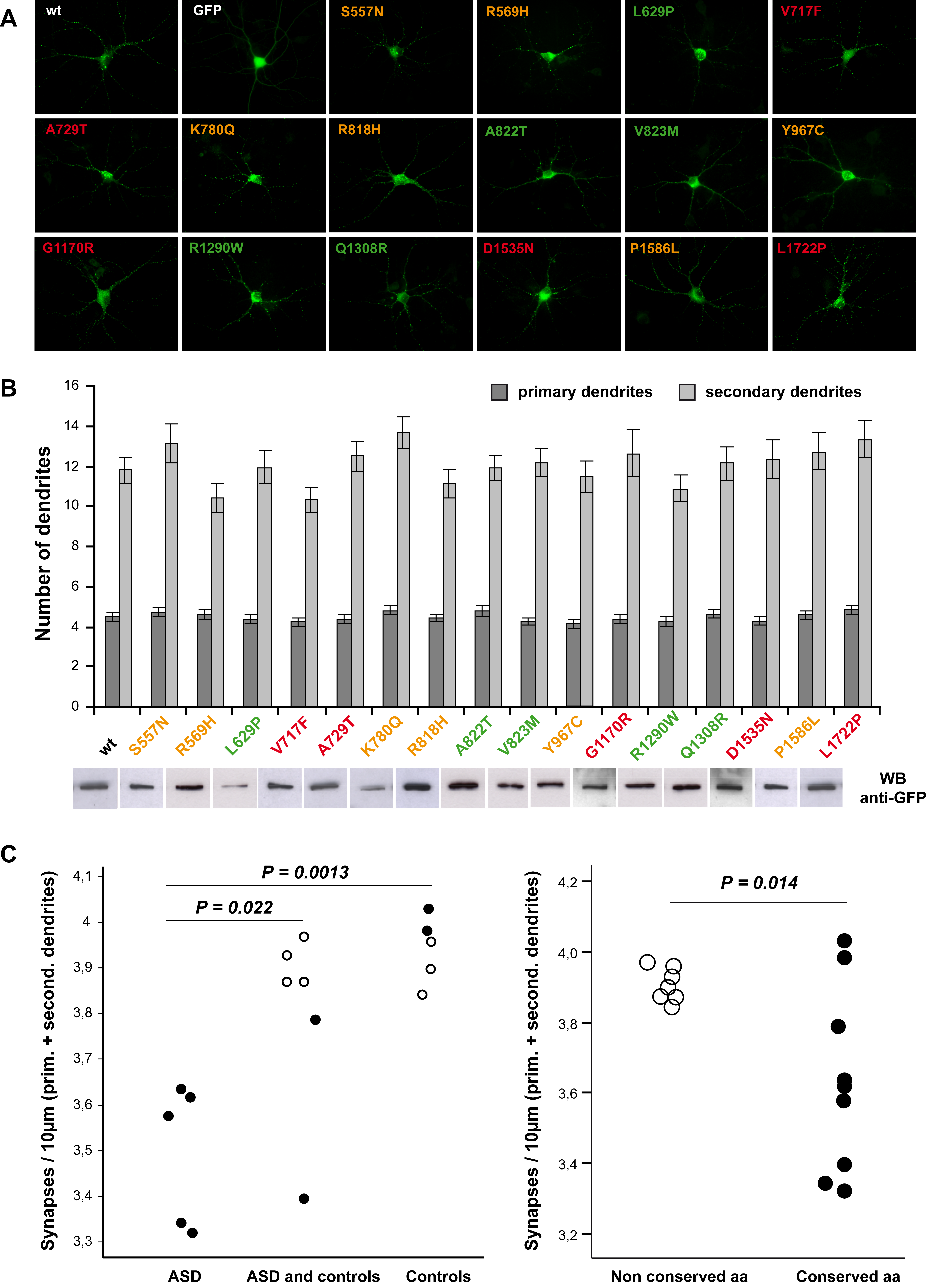

Supplement: Figure S4 — Functional analysis of shank2 mutations in cultured hippocampal neurons. A. Rat hippocampal neurons transfected with wild-type or mutated ProSAP1A/SHANK2-EGFP cDNA constructs were detected by EGFP expression. B. The quantification of dendrite number was performed on 20 transfected hippocampal neurons per construct from at least 3 independent experiments. No mutation affected the number of dendrites per neuron (Bars represent mean ± SD, Mann-Whitney U test and Kolmogorov-Smirnov Z test; no significant differences; nWT = 20, nmut = 20). Western blot analysis of ProSAP1A/SHANK2-EGFP cDNA constructs revealed similar sizes of WT fusion ProSAP1A/SHANK2-EGPF protein and the constructs carrying the mutations. C. The variants identified in patients with ASD were associated with reduced synaptic density compared with those identified in controls. D. The variants affecting conserved amino acids in other SHANK proteins were associated with reduced synaptic density compared with those affecting non conserved amino acids. Variations represented in red were specific to ASD, those in orange were shared by ASD and controls, and those in green were specific to controls. (TIF) [file pgen.1002521.s004.tif]
