# Supplementary material for: Genetic and Functional Analyses of SHANK2 Mutations Suggest a Multiple Hit Model of Autism Spectrum Disorders
Source: PLoS Genet. 2012 Feb 9;8(2):e1002521. doi: 10.1371/journal.pgen.1002521 (PMC3276563; doi:10.1371/journal.pgen.1002521)
Supplement: Table S1 — Description of the population of patients with ASD analyzed in this study. (DOC) [file pgen.1002521.s005.doc]

**Table S1. Description of the population of patients with ASD analyzed in this study.**

|  |  |  | **Sex** | | | **Intellectual disability** | | |
| --- | --- | --- | --- | --- | --- | --- | --- | --- |
|  |  | **Total** | **Male** | **Female** | **Unknown** | **IQ>70** | **IQ<70** | **Unknown** |
| **Mutation screening** | Autism | n=255 | 203 | 52 | 0 | 59 | 168 | 28 |
|  | Atypical autism | n=50 | 36 | 12 | 2 | 7 | 14 | 29 |
|  | Asperger syndrome | n=11 | 9 | 2 | 0 | 10 | 0 | 1 |
|  | **Total** | **n=316** | **248** | **66** | **2** | **76** | **182** | **58** |
| **CNV screening**  **(Illumina 1M Duo)** | Autism | n=57 | 45 | 12 | 0 | 7 | 39 | 11 |
|  | Atypical autism | n=15 | 7 | 2 | 6 | 1 | 0 | 14 |
|  | Asperger syndrome | n=32 | 27 | 5 | 0 | 7 | 2 | 23 |
|  | **Total** | **n=104** | **79** | **19** | **6** | **15** | **41** | **48** |
| **Mutation & CNV screening** | Autism | n=114 | 83 | 31 | 0 | 27 | 87 | 0 |
|  | Atypical autism | n=5 | 4 | 1 | 0 | 1 | 4 | 0 |
|  | Asperger syndrome | n=37 | 28 | 9 | 0 | 36 | 1 | 0 |
|  | **Total** | **n=156** | **115** | **41** | **0** | **64** | **92** | **0** |
| **Total** | Autism | n=426 | 331 | 95 | 0 | 93 | 294 | 39 |
|  | Atypical autism | n=70 | 47 | 15 | 8 | 9 | 18 | 43 |
|  | Asperger syndrome | n=80 | 64 | 16 | 0 | 53 | 3 | 24 |
|  | **Total** | **n=576** | **442** | **126** | **8** | **155** | **315** | **106** |
