# Supplementary material for: Genetic and Functional Analyses of SHANK2 Mutations Suggest a Multiple Hit Model of Autism Spectrum Disorders
Source: PLoS Genet. 2012 Feb 9;8(2):e1002521. doi: 10.1371/journal.pgen.1002521 (PMC3276563; doi:10.1371/journal.pgen.1002521)
Supplement: Table S2 — Clinical description of the patients carrying predicted deleterious SHANK2 variations. Abbreviations: ADI-R, Autism Diagnosis Interview-Revised; ASD, autism spectrum disorder; CT, computed tomography; ERG, electroretinogram; F, female; FSIQ, full scale IQ; HC, head circumference; K-ABC, Kaufman Assessment Battery for Children; M, male; MRI, magnetic resonance imaging; MZ, monozygotic; NA, not available; ND, not done; PDD-NOS, pervasive developmental disorder not otherwise specified; PET, positron emission tomography; PIQ, performance IQ; PEP-R, Psychoeducational Profile-Revised; VIQ, verbal IQ; WISC-III, Wechsler Intelligence Scale for Children-Third Edition; WPPSI, Wechsler Preschool and Primary Scale of Intelligence; IQ, intellectual quotient. (DOC) [file pgen.1002521.s006.doc]

**Table S2. Clinical description of the patients carrying predicted deleterious *SHANK2*** variations

| **Subject** | **AU038_3** | **AU118_3** | **AU079_3** | **AU005_6** | **AU017_3** | **AU028_3** | **AU112_3** | **C_37_3** |
| --- | --- | --- | --- | --- | --- | --- | --- | --- |
| ***SHANK2* genetic findings** | Del Ex4-Ex15 | R185Q | V717F - P1586L | A729T | G1170R | D1535N | L1722P | R598L |
| **Inheritance** | *de novo* | mother | father - mother | mother | mother | mother | father | NA |
| **Sex** | M | M | M | F | M | M | M | M |
| **Ethnicity** | Caucasian (European) | Caucasian (European) | Caucasian (Iran) | Caucasian (European) | Father European, mother North Africa | Caucasian (Scandinavia) | father Iran, mother Scandinavia | Italian |
| **Family type** | singleton | singleton | singleton | sib-pair (sister of AU005.1_5) | singleton | singleton | Singleton (MZ twins, both affected) | singleton |
| **ASD diagnosis** | autism | autism | autism | autism | autism | autism | autism | autism |
| **Cognitive level** |  |  |  |  |  |  |  |  |
| Age at evaluation (y) | 11.05 | 3.6 | 6.01 | 6.09 | 36.05 | 11.09 | 2.0 | 5.03 |
| Instrument | K-ABC | PEP-R | WPPSI | Vineland | Vineland | WISC-III | WPPSI-R | PEP-R |
| IQ or Developmental age (y) | composite score 40 | 1.3-4.0 | FSIQ 90 | 2.1-3.0 | 0.9-2.5 | FSIQ 63 | PIQ 78, VIQ 67, FSIQ 69 | developmental quotient = 33 |
| **Pregnancy and delivery** | normal pregnancy and delivery, at term; height 48 cm, weight 2500 g, HC 31 cm | normal pregnancy, long delivery, Apgar 6/10, HC 34 cm | normal pregnancy and delivery; HC 37 cm | normal pregnancy and delivery, born at 38 weeks | normal pregnancy, Cesarean section for lack of progress and fetal distress | normal pregnancy and delivery; height 46 cm, weight 2800 g, HC 34 cm | mother had latent diabetes during pregnancy, born at 42 weeks, neonatal hypoglycemia; weight 5280 g | first-born, in the first two months pharmacologically treated with ritodrine hydrochloride, born at 40 weeks and 4 days, weight: 3600 g, height: 51 cm |
| **Developmental stages** |  |  |  |  |  |  |  |  |
| Age of sitting (m) | 6 | normal | 8 | NA | 9 | 6 | normal | NA |
| Age of walking (m) | 18 | 13 | 11 | 12 | 11 | 14 | 13 | 14 |
| Age of first words (m) | 48 | 72 | 18 | no language at 6.09 y | non verbal | 42 | 36 | 18 |
| Age of first sentences (m) | 78 | non verbal | 48 | no language | non verbal | 48 | 40 | 60 |
| Regressive episode during development (age) | no | no | no | NA | yes (24 m) | no | no | NA |
| **Medical Conditions** |  |  |  |  |  |  |  |  |
| History of seizures | no | no | no | no | yes | no | no | no |
| Age at first seizure (y) |  |  |  |  | 12 |  |  |  |
| Current anti-epileptic treatment |  |  |  |  | yes |  |  |  |
| Type of seizure |  |  |  |  | generalized tonico-clonic seizures |  |  |  |
| Other comorbid conditions | inguinal hernia, operated at 2 mo of age; hypermetropia and astigmatism | hearing deficit detected at 3 y, treated with hearing aid for 1 year | no | explosive behavior, overactivity |  | hearing deficit detected at 1 y, treated with hearing aid; audiogram at 4 y normal | inguinal hernia operated at 2 mo of age | hyperactivity, tics |

**Table S2.** Continued.

| **Subject** | **AU038_3** | **AU118_3** | **AU079_3** | **AU005_6** | **AU017_3** | **AU028_3** | **AU112_3** | **C_37_3** |
| --- | --- | --- | --- | --- | --- | --- | --- | --- |
| **Clinical Examination** | prominent chin, no other dysmorphic features, normal neurological exam | clinodactyly 5th fingers, prominent ears, no dysmorphic features, normal neurological exam | no dysmorphic features, normal physical and neurological exam | slight syndactyly of 2°-3° toes bilaterally, no dysmorphic features; at 6.09 y height 109 cm (<3rd percentile), weight 20 kg. Dysdiadochokinesis, hypotonia, good motor skills | macroglossia, short philtrum, thin upper lip, troncular obesity, normal physical and neurological exam | no dysmorphic features, normal physical and neurological exam | mild strabismus, no dysmorphic features, +2 SD to +3 SD in weight and length | no dysmorphic features, normal physical and neurological exam, at 4.01 y height >97°pc, weight > 97°pc and head circumference = 75°pc |
| **Family history** | Negative | Negative | Father stammering during childhood | Two older brothers language delay, one affected older sister; mother anxiety; maternal cousin epilepsy | Maternal uncle schizophrenia | Paternal uncle schizophrenia; paternal aunt depression; maternal uncle suicide; maternal cousin schizophrenia, suicide | Negative | Mother and maternal uncle: anxiety and depressive disorder; Maternal uncle: seizure; paternal cousin: language delay |
| **Brain imaging** | ND | MRI at 10 y: small temporal arachnoid cyst with mild mass effect | normal MRI | normal MRI | ND | normal CT | ND | normal MRI |
| **Other exams** | karyotype, fragile X, metabolic screening and EEG normal | karyotype, fragile X, metabolic screening and ERG normal | karyotype, fragile X, metabolic screening and EEG normal | karyotype, fragile X, metabolic screening and EEG normal | karyotype, fragile X, and metabolic screening normal | karyotype, fragile X, and metabolic screening normal | karyotype: 46,XY,der(4)t(4;acro p)(p16.3;acro p) de novo, subtelomeric 4p deletion and 4 Mb 4p duplication | karyotype, fragile X, ECG and EEG normal |
