# Supplementary material for: Genetic and Functional Analyses of SHANK2 Mutations Suggest a Multiple Hit Model of Autism Spectrum Disorders
Source: PLoS Genet. 2012 Feb 9;8(2):e1002521. doi: 10.1371/journal.pgen.1002521 (PMC3276563; doi:10.1371/journal.pgen.1002521)
Supplement: Table S3 — Frequency of SHANK2 R818H variation in 3250 patients with ASD and 2013 controls. OR, odds ratio; P, p-value. (DOC) [file pgen.1002521.s007.doc]

**Table S3. Frequency of *SHANK2* R818H variation in 3250 patients with ASD and 2013 controls.**

|  | ASD (n=3250) | | | Controls (n=2013) | | | Fisher's exact test 2-sided | |
| --- | --- | --- | --- | --- | --- | --- | --- | --- |
|  | R818H/+ | +/+ | Allelic frequency (%) | R818H/+ | +/+ | Allelic frequency (%) | P | OR |
| France | 4 | 579 | 0.34 | 0 | 508 | 0.00 | 0.13 | ∞ |
| Sweden | 2 | 168 | 0.59 | 7 | 293 | 1.17 | 0.50 | 0.50 |
| United Kingdom | 6 | 1004 | 0.30 | 1 | 292 | 0.17 | 1 | 1.74 |
| Italy | 5 | 285 | 0.86 | 2 | 236 | 0.42 | 0.47 | 2.07 |
| Finland | 11 | 186 | 2.79 | 11 | 256 | 2.06 | 0.51 | 1.38 |
| Portugal | 0 | 481 | 0.00 | 0 | 70 | 0.00 | 1 | 0 |
| Germany | 4 | 516 | 0.38 | 6 | 331 | 0.89 | 0.20 | 0.43 |
| **Total** | **32** | **3218** | **0.49** | **27** | **1986** | **0.67** | 0.28 | 0.73 |
| Berkel et al [18] | 1 | 396 | 0.12 | 4 | 655 | 0.30 | 0.66 | 0.41 |
