# Supplementary material for: Genetic and Functional Analyses of SHANK2 Mutations Suggest a Multiple Hit Model of Autism Spectrum Disorders
Source: PLoS Genet. 2012 Feb 9;8(2):e1002521. doi: 10.1371/journal.pgen.1002521 (PMC3276563; doi:10.1371/journal.pgen.1002521)
Supplement: Table S4 — Frequency of SHANK2 R818H variation in 948 individuals from the Human Genome Diversity Panel. (DOC) [file pgen.1002521.s008.doc]

**Table S4. Frequency of *SHANK2* R818H variation in 948 individuals from the Human Genome Diversity Panel.**

| **HGDP** |  | **R818H/+** | **+/+** | **Allelic frequency (%)** |
| --- | --- | --- | --- | --- |
| Europe (n=161) | Russia | 4 | 41 | 4.87 |
|  | France | 0 | 51 | 0.00 |
|  | Orkney Islands | 1 | 14 | 3.57 |
|  | Italy | 0 | 50 | 0.00 |
| Subsaharan Africa (n=101) | Central African Republic | 0 | 25 | 0.00 |
|  | Democratic Republic of Congo | 0 | 12 | 0.00 |
|  | Namibia | 0 | 5 | 0.00 |
|  | South Africa | 0 | 7 | 0.00 |
|  | Kenya | 0 | 10 | 0.00 |
|  | Nigeria | 0 | 21 | 0.00 |
|  | Senegal | 0 | 21 | 0.00 |
| North Africa (n=28) | Algeria | 0 | 28 | 0.00 |
| Asia (n=434) | China | 0 | 179 | 0.00 |
|  | Cambodia | 0 | 9 | 0.00 |
|  | Japan | 0 | 29 | 0.00 |
|  | Siberia | 0 | 24 | 0.00 |
|  | Pakistan | 0 | 193 | 0.00 |
| Middle East (n=134) | Israel | 0 | 134 | 0.00 |
| Oceania (n=29) | Bougainville | 0 | 13 | 0.00 |
|  | New Guinea | 0 | 16 | 0.00 |
| America (n=61) | Mexico | 0 | 34 | 0.00 |
|  | Colombia | 0 | 6 | 0.00 |
|  | Brazil | 0 | 21 | 0.00 |
| **n=948** | **Total** | **5** | **943** | **0.26** |
