# Supplementary material for: Genetic and Functional Analyses of SHANK2 Mutations Suggest a Multiple Hit Model of Autism Spectrum Disorders
Source: PLoS Genet. 2012 Feb 9;8(2):e1002521. doi: 10.1371/journal.pgen.1002521 (PMC3276563; doi:10.1371/journal.pgen.1002521)
Supplement: Table S5 — Evolutionary conservation of SHANK2 protein sequence. Variations identified only in patients with ASD, only in controls or shared by patients and controls are indicated in red, green and orange, respectively. Hu, human; Ch, chimpanzee; Ma, macaque; Ra, rat; Mo, mouse; Ck, chicken; Zn, zebrafinsh; Zf, zebrafish; Xe, xenopus. (DOC) [file pgen.1002521.s009.doc]

Table S5. Evolutionary conservation of SHANK2 protein sequence.

| Mutations | **R174C** | **R185Q** | **R405W/T410M/R415W** | **D432N** | **R443C** | **M453V** | **E514G** | **S557N** | **R569H** | **P587S** |
| --- | --- | --- | --- | --- | --- | --- | --- | --- | --- | --- |
| SHANK2_Hu  SHANK2_Ch  SHANK2_Ma  SHANK2_Ra  SHANK2_Mo  SHANK2_Ck  SHANK2_Zn  SHANK2_Zf  SHANK2_Xe  SHANK3_Hu  SHANK1_Hu | HIQH**R**LVEK  HIQH**R**LVEK  HIQH**R**SVEK  HTQH**R**SVEK  YVQL**G**TSDK  HVHH**L**SVEK  ----**-**----  YVQQ**R**CVDK  YIQL**Q**NIEK  YVQL**H**STDK  YVQL**G**TSDK | KMLD**R**GLDP  KMLD**R**GLDP  KMLD**R**GLDP  KLLD**R**GLDP  RLLD**K**GLDP  KMLD**R**GLDP  TLLD**R**GLDE  RFLE**K**GLDP  RLLE**R**GLDP  RLLD**K**GLDP  RLLD**K**GLDP | SNRR**R**RPPN**T**LAAP**R**VLLR  SNRR**R**RPPN**T**LAAP**R**VLLR  SNRR**R**RPPN**T**LAAP**R**VLLR  SNRR**R**RPPN**T**LAAP**R**VLLR  SNRR**R**RPPN**T**LAAP**R**VLLR  SNRR**R**RPPS**T**LAAP**R**ILLR  SNRR**R**RPPS**T**LAAP**R**ILLR  TGRS**R**QGRS**T**LAAP**R**VLLR  TKRR**R**VSSV**G**LSSP**R**FLQR  AKRR**R**LAPS**G**LASP**R**PLQR  AARR**R**GPGT**G**LTVP**P**ALLR | ASAP**D**WAVC  ASAP**D**WAVC  ASAP**E**WAVC  AGAP**E**WAVC  AGAP**E**WAVC  INIP**E**WSAS  INLP**E**WSAS  VNKL**S**LSPS  VNLP**E**WSAS  GEAQ**-**-PAA  VFSA**P**GAAS | ATSH**R**SLSP  ATSH**R**SLSP  ATSH**R**SLSP  ATSH**R**SLSP  ATSH**R**SLSP  ASSH**R**SLSP  ASSH**R**SLSP  ASPL**R**SLSP  ASLH**R**SLSP  GPSL**R**SLPH  APGP**T**SGSQ | LLQQ**M**PSKP  LLQQ**M**PSKP  LLQQ**M**PSKP  LLQQ**T**PSKP  LLQQ**T**PSKP  LLQQ**M**QNNP  LLQQ**M**QNNP  HPQQ**M**QNSP  LLQQ**M**QNNP  LLLQ**R**LQEE  QSQP**S**APTT | LSAF**E**YPGP  LSAF**E**YPGP  LSAF**E**YPGP  LSTF**E**YPGP  ----**-**--SP  ----**-**--SP  ----**-**--SP  ----**-**SHGP  ----**-**--PP  PPAP**P**PRGP  GGSL**G**SRGR | VKVL**S**IGEG  VKVL**S**IGEG  VKVL**S**IGEG  VKVL**S**IGEG  VKVL**S**IGEG  VKVL**S**IGEG  VKVL**S**IGEG  VKVL**S**IGEG  IRVL**S**IGEG  VKVL**S**IGEG  IKVL**S**IGEG | EGSA**R**GHIG  EGSA**R**GHIG  EGSA**R**GHIG  EGSA**R**GHIG  EGSA**R**GHIG  EGST**R**GHIG  EGST**R**GHIG  EGSA**R**GNVG  EGNA**R**GHVG  EGTV**K**GRTG  EGQV**K**GRVG | VQCK**P**RDSQ  VQCK**P**RDSQ  VQMR**Q**HD--  VQCK**P**RD--  -VQC**K**PRD-  VQCK**P**NE--  -VQC**K**PNE-  VQMR**Q**YDPR  IQPK**P**SD—  **V**QMR**Q**HDTR  **V**ANR**S**QESK |

Table S5. continued.

| Mutations | **R598L** | **L629P** | **V717F** | **A729T** | **K780Q** | **T796N/P801T** | **R818H** | **A822T/V823M/T825M** | **R841X** | **Y967C** |
| --- | --- | --- | --- | --- | --- | --- | --- | --- | --- | --- |
| SHANK2_Hu  SHANK2_Ch  SHANK2_Ma  SHANK2_Ra  SHANK2_Mo  SHANK2_Ck  SHANK2_Zn  SHANK2_Zf  SHANK2_Xe  SHANK3_Hu  SHANK1_Hu | TRAD**R**SKKL  TRAD**R**SKKL  PR-D**R**TKRL  SQAD**R**SKKL  -SQD**R**SKKL  SKPD**R**TKKL  -SKD**R**TKKL  TRED**R**NKRL  GRPD**R**TKKL  TRED**R**TKRL  SRSD**K**AKRL | KTVV**L**QKKD KTVV**L**QKKD KTVV**L**QKKD KTVV**L**QKKD KTVV**L**QKKD KAVV**L**QKKD KAVV**L**QKKD KTVV**L**QKKD KTVV**L**QKKD  KVAV**L**QKRD KTVL**L**QKKD | VLKV**V**TVTR  VLKV**V**TVTR  VLKV**V**TVTR  VLKV**V**TVTR  ILKV**V**TVTR  VLKV**V**TVTR  VLKV**V**TVTR  VMKV**V**SVTR  VLKV**V**TVTR  VMKV**V**SVTR  MVKV**V**MVTR | PDDT**A**RKKA  PDDT**A**RKKA  PDDT**A**RKKA  PDDT**A**RKKA  PDDT**A**RKKA  PDDT**A**RKKA  PDDT**A**RKKA  TGDV**V**RKKA  PDDT**A**RKKA  -EDG**A**RRRA  MDEA**V**HKKA | VPAS**K**PSRA  VPAS**K**PSRA  VPAS**K**PSRA  VPAS**K**PSRT  VPAS**K**PSRT  VPVS**K**PSRI  VPVS**K**PSRI  VPPQ**K**P--I  GPIS**K**PLRP  AAAA**E**PTLR  VYQM**A**LNKL | PRVA**T**IKQR**P**SSRC  PRVA**T**IKQR**P**SSRC  PRVA**T**IKQR**P**SSRC  SRVA**T**IKQR**P**TSRC  SRVA**T**IKQR**P**TSRC  SRVA**T**IKQR**P**SSRC  SRVA**T**IKQR**P**SSRC  YRAA**T**VKQR**P**TSRR  SRIA**T**VKQR**P**TSRC  SRAA**T**VKQR**P**TSRR  QQTI**S**ASES**P**GPGG | SVYE**R**QGIA  SVYE**R**QGIA  SVYE**R**QGIA  SVYE**R**QGIA  SVYE**R**QGIA  SMYE**R**QGIA  SMYE**R**QGIA  LCNS**Q**DTAV  SMYD**R**QGIA  SLFE**R**QGLP  GFFA**T**ESSF | RQGI**AV**M**T**PT RQGI**AV**M**T**PT RQGI**AV**M**T**PT RQGI**AV**M**T**PT RQGI**AV**M**T**PT RQGI**AV**M**T**PT RQGI**AV**M**T**PT QDTA**VD**S**S**PQ RQGI**AV**I**P**PT  RQGL**PG**P**E**-K TESS**FD**P**H**HR | LGIP**R**GTMR  LGIP**R**GTMR  LGIP**R**GTMR  LGLP**R**GTMR  LGLP**R**GTMR  LGIP**R**GTMR  LGIP**R**GTMR  LGLP**R**GTIR  LGIP**R**GTMR  -GIP**R**----  FLPP**G**---- | SEDL**Y**SRNA  SEDL**Y**SRNA  SEDL**Y**SRNA  SEDV**Y**SRSP  SEDV**Y**SRSP  SEDL**Y**SRSA  SEDL**Y**NRSA  SEG-**Y**NHSP  SEEL**Y**NSNM  APRP**P**PAAT  ---P**P**RLAL |

Table S5. continued.

| Mutations | **E1162K** | **G1170R** | **R1290W** | **Q1308R** | **P1367A** | **V1376I** | **Dup(LP) 1387-1388** | **A1429S** | **P1456T** | **T1506M** | **D1535N** |
| --- | --- | --- | --- | --- | --- | --- | --- | --- | --- | --- | --- |
| SHANK2_Hu  SHANK2_Ch  SHANK2_Ma  SHANK2_Ra  SHANK2_Mo  SHANK2_Ck  SHANK2_Zn  SHANK2_Zf  SHANK2_Xe  SHANK3_Hu  SHANK1_Hu | VGGA**E**ASAP  VGGA**E**ASAQ  VGGA**E**ASAQ  LGGG**E**AGAQ  LGGG**E**AGAQ  -NSS**E**PSNQ  -NSS**E**PSNQ  -NSP**E**PTM-  -TNN**D**SSHQ  LG--**-**-TGP  LR--**-**-LES | PGEA**G**RPLN  QGEA**G**RPLN  QGEA**G**RPLN  QGEA**G**GPLS  QGEA**G**GPLS  QSDA**-**RTLN  QSDS**-**RTLN  PGEN**G**AVLT  PKRL**P**PRTG  P-TA**G**RDLL  SAGS**G**AGYG | LGRD**R**KGDD LGRD**R**KGDD LGRD**R**KGDD LSKD**R**RADD LGKD**R**RADD AGKE**K**KAEE AGKE**K**KAEE LGKE**K**RPEE SSKE**K**RQGE  VPRE**E**RKS-  LGSQ**E**KSLP | DTSQ**Q**KSAG DTSQ**Q**KSAG DTSQ**Q**KSAG DTAQ**Q**KSAG DTAQ**Q**KSAG DTSQ**Q**KSAG DTSQ**Q**KSAG DTSQ**Q**KSAG DTSQ**Q**KTAG  DTSL**Q**RPAGPTAP**G**VGPL | AP-E**P**TTVP  AP-E**P**TTVP  AP-E**P**TTVP  AP-E**P**AAAP  AP-E**P**AVAP  VP-E**P**PASP  AP-E**P**PASP  KA-L**S**AA-P  LG-T**P**AA-P  AQ-P**P**GGTP  GPGV**P**PPSP | GRTI**V**AVGS  GRTI**V**AVGS  GRTI**V**AVGS  GRTI**V**AAGS  GRTI**V**AAGS  CKTI**V**AASS  CKTI**V**AASS  GKTI**I**TVSS  NKAI**V**SVCS  ADAG**P**GQGS  RRSV**P**PSPT | EAVI**LP**FRIP  EAVI**LP**FRIP  EAVI**LP**FRIP  EAVI**LP**FRIP  EAVI**LP**FRIP  DPVI**LP**FRIP  DPVI**LP**FRIP  EPVK**LP**FGIP  EPVI**LP**FRIP  EEPE**LV**FAVN  EENG**LP**LLVL | DRA-**A**SVPA  DRA-**A**SVPA  DRA-**A**SVPA  DRA-**A**SVPA  DRA-**A**SVPA  DRS-**V**PASA  DRA-**A**PGSA  DQA-**-**--GS  DASQ**M**PPAT  --RI**G**LVPP  ATPL**P**PVPP | NSSQ**P**TNSA  NSSQ**P**TNSA  NSSQ**P**TNSA  NSSQ**P**ANST  NSSQ**P**ANST  VTSQ**P**TNSL  --SQ**P**ANSL  YPAL**G**GGHA  NPNV**A**SNSI  PSPT**T**VPSP  SYDS**E**VATL | HHLE**T**TSTI  HHLE**T**TSTI  HHLE**T**TSTI  HHLE**T**TSTI  HHLE**T**TSTI  HHLE**T**TSTI  HHLE**T**TSTI  PQLE**T**TSTI  HHLE**T**TSTI  PHLE**T**TSTI  HPLE**T**ISSA | TVYA**D**GQAF  TVYA**D**GQAF  TVYA**D**GQAF  TVYA**D**GQAF  TVYA**D**GQAF  TVYA**D**GQTF  TVYA**D**GQTF  TVYA**D**GQAF  TVYA**D**GQAF  TSFA**D**GHTF  VAYL**D**GQAF |

**Table S5. continued.**

| Mutations | **P1586L** | **I1664T** | **D1696N** | **M1717I** | **L1722P** | **A1729T** |
| --- | --- | --- | --- | --- | --- | --- |
| SHANK2_Hu  SHANK2_Ch  SHANK2_Ma  SHANK2_Ra  SHANK2_Mo  SHANK2_Ck  SHANK2_Zn  SHANK2_Zf  SHANK2_Xe  SHANK3_Hu  SHANK1_Hu | PPPP**P**GSAQ  PPPP**P**GSAQ  PPPP**P**GSAQ  PPPP**P**GGAQ  PPPP**P**GSAQ  PPPP**P**ISAQ  PPPP**P**ISAP  PPPP**P**GSIP  PPPL**P**LSIQ  HAAS**A**GLAS  TAGV**A**GGPV | RSPE**I**MSTI  RSPE**I**MSTI  RSPE**I**MSTI  RSPE**V**MSTV  RSPE**V**MSTV  RSTE**V**MSTV  RSTE**V**MSTV  RGTD**A**LSTV  RNQD**G**VSLI  KSPI**A**AARL  TSSL**Q**RQRL | SRPP**D**YESR  SRPP**D**YESR  SRPP**D**YESR  SRPP**D**YESR  SRPP**D**YESR  SRSP**D**YDSR  SRSP**D**YDSR  CRQA**E**---S  NRTP**E**FEGR  ---VRPSGR  LQGVEFEMR | SPTE**M**NKET  SPTE**M**NKET  SPTE**M**NKET  SPTE**L**SKEI  SPTE**L**SKEI  SPTE**I**NKDI  SPTE**I**NKDI  SPPD**S**GLSC  SPAE**I**TRDI  PASL**E**RVEG  LPAS**E**HKVS | NKET**L**PAPL  NKET**L**PAPL  NKET**L**PTPL  SKEI**L**PTPL  SKEI**L**PTPT  NKDI**M**PAPL  NKDI**L**PAPL  DPYS**Q**PFPN  EIIR**D**IKPG  RVEG**L**GAGA  HKVS**L**PSGP | --SA**A**TASP  --SA**A**TASP  --SA**A**AASP  --SA**A**AASP  PPSA**T**AASP  TASA**S**ASSP  TASA**S**ASSP  T---**-**-SAT  ----**-**-SAP  GAGG**A**GRPF  GPLY**P**GLFD |
