# Supplementary material for: Genetic and Functional Analyses of SHANK2 Mutations Suggest a Multiple Hit Model of Autism Spectrum Disorders
Source: PLoS Genet. 2012 Feb 9;8(2):e1002521. doi: 10.1371/journal.pgen.1002521 (PMC3276563; doi:10.1371/journal.pgen.1002521)
Supplement: Table S6 — List of all CNVs observed in ASD patients carrying a de novo deletion of SHANK2. The 6319_3 and 5237_3 patients were described by the AGP [9]. QSNP, QuantiSNP; PCNV, PennCNV; IP, iPattern. (DOC) [file pgen.1002521.s010.doc]

**Table S6. List of all CNVs observed in ASD patients carrying a *de novo* deletion of *SHANK2***.

|  | **chr** | **Start position** | **Stop position** | **Length (bp)** | **CNV** | **Detection Method** | **RefSeq Genes** | **Region** | **inheritance** |
| --- | --- | --- | --- | --- | --- | --- | --- | --- | --- |
| AUGB038_3 | 1 | 25461733 | 25519520 | 57788 | loss | QSNP, PCNV, SnipPeep | RHD | Exon | Father |
| AUGB038_3 | 1 | 199008729 | 199031936 | 23208 | gain | QSNP, PCNV, SnipPeep | CAMSAP1L1 | Intron | Father |
| AUGB038_3 | 1 | 246812825 | 246859583 | 46759 | loss | QSNP, PCNV, SnipPeep | OR2T10-11 | Exon | Mother |
| AUGB038_3 | 2 | 38805170 | 38818035 | 12866 | gain | QSNP, PCNV, SnipPeep | GALM | Exon | Mother |
| AUGB038_3 | 6 | 79034386 | 79090197 | 55812 | loss | QSNP, PCNV, SnipPeep | - | - | Father |
| AUGB038_3 | 6 | 140536841 | 140667771 | 130931 | loss | QSNP, PCNV, SnipPeep | - | - | Father |
| AUGB038_3 | 11 | 70077507 | 70498707 | 421201 | loss | QSNP, PCNV, SnipPeep | SHANK2 | Exon | *De novo* |
| AUGB038_3 | 14 | 19207427 | 19493705 | 286279 | gain | QSNP, SnipPeep | OR4Q3, OR4M1, OR4N2, OR4K2, OR4K5, OR4K1 | Exon | Father |
| AUGB038_3 | 15 | 28723577 | 28773343 | 49767 | loss | QSNP, PCNV, SnipPeep | ARHGAP11B | Exon | Mother |
| AUGB038_3 | 15 | 29807211 | 30303265 | 496055 | gain | QSNP, PCNV, SnipPeep | CHRNA7 | Exon | Mother |
| AUGB038_3 | 16 | 34454657 | 34607434 | 152778 | gain | QSNP, PCNV, SnipPeep | - | - | Mother |
| 6319_3 | 1 | 91329245 | 91351436 | 22192 | loss | IP, QSNP|PCNV | - | - | Mother |
| 6319_3 | 1 | 103907158 | 104135740 | 228583 | gain | IP,QSNP|PCNV | AMY2B, LOC648740, AMY2A, AMY1A, AMY1C, AMY1B | exon | Mother |
| 6319_3 | 2 | 89390395 | 89929625 | 539231 | loss | IP,QSNP|PCNV | - | - | Father |
| 6319_3 | 3 | 2392491 | 2462197 | 69707 | loss | IP,QSNP|PCNV | CNTN4 | intron | Mother |
| 6319_3 | 3 | 53003023 | 53029767 | 26745 | loss | IP,QSNP|PCNV | SFMBT1 | intron | Father/Mother |
| 6319_3 | 4 | 21657735 | 21682866 | 25132 | loss | IP,QSNP|PCNV | - | - | Mother |
| 6319_3 | 4 | 69064675 | 69163188 | 98514 | loss | IP,QSNP|PCNV | UGT2B17 | exon | Father/Mother |
| 6319_3 | 5 | 140206092 | 140226471 | 2038 | loss | IP,QSNP|PCNV | PCDHA2-8  PCDHA9-10 | intron exon | Father |
| 6319_3 | 6 | 79029649 | 79090197 | 60549 | loss | IP,QSNP|PCNV | - | - | Mother |
| 6319_3 | 7 | 47272494 | 47290661 | 18168 | gain | IP,QSNP|PCNV | TNS3 | exon | Mother |
| 6319_3 | 8 | 9093118 | 9099900 | 6783 | loss | IP,QSNP|PCNV | - | - | Father |
| 6319_3 | 9 | 105920215 | 106334543 | 414329 | gain | IP,QSNP|PCNV | OR13C4, SMC2, OR13F1 | exon | Mother |
| 6319_3 | 11 | 25660411 | 25677282 | 16872 | loss | IP,QSNP|PCNV | - | - | Father |
| 6319_3 | 11 | 70119917 | 70187872 | 67956 | loss | IP,QSNP|PCNV | SHANK2 | exon | *De novo* |
| 6319_3 | 12 | 14093522 | 14212591 | 11907 | loss | IP,QSNP|PCNV | - | - | Mother |
| 6319_3 | 15 | 20305097 | 20773130 | 468034 | loss | IP,QSNP|PCNV | NIPA2, NIPA1, CYFIP1, TUBGCP5, WHDC1L1 | exon | Father |
| 6319_3 | 15 | 32459510 | 32625184 | 165675 | loss | IP,QSNP|PCNV | GOLGA8A, GOLGA8B | exon | Mother |
| 6319_3 | 15 | 95616714 | 95757068 | 140355 | loss | IP,QSNP|PCNV | - | - | Father |
| 6319_3 | 16 | 16154972 | 16168752 | 13781 | loss | IP,QSNP|PCNV | ABCC6 | exon | Father |
| 6319_3 | 18 | 64897188 | 64906488 | 9301 | loss | IP,QSNP|PCNV | - | - | Father |
| 6319_3 | X | 134583959 | 134628030 | 44072 | gain | IP,QSNP | - | - | *De novo* |
| 5237_3 | 1 | 103954623 | 103961238 | 6616 | loss | IP,QSNP | - | - | Father |
| 5237_3 | 1 | 167493526 | 167507362 | 13837 | loss | IP,QSNP|PCNV | NME7 | intron | Mother |
| 5237_3 | 2 | 34556561 | 34580068 | 23508 | loss | IP,QSNP|PCNV | - | - | Father/Mother |
| 5237_3 | 3 | 75502426 | 75679402 | 176977 | loss | IP,QSNP|PCNV | FAM86D | exon | Mother |
| 5237_3 | 3 | 174722147 | 174771975 | 49829 | gain | IP,QSNP|PCNV | NLGN1 | intron | Mother |
| 5237_3 | 4 | 57298439 | 57316600 | 18162 | loss | IP,QSNP|PCNV | - | - | Mother |
| 5237_3 | 5 | 8756085 | 8800106 | 44022 | loss | IP,QSNP|PCNV | - | - | Father |
| 5237_3 | 6 | 194597 | 326217 | 131621 | loss| | IP,QSNP|PCNV | DUSP22 | exon | *De novo* |
| 5237_3 | 6 | 10575739 | 10636780 | 61042 | loss | IP,QSNP|PCNV | GCNT2 | exon | Father |
| 5237_3 | 6 | 32593470 | 32674376 | 80907 | gain | IP,QSNP|PCNV | HLA-DRB1, HLA-DRB6, HLA-DRB5 | exon | Father/Mother |
| 5237_3 | 6 | 112680473 | 112778848 | 98376 | gain | IP,QSNP|PCNV | RFPL4B, LAMA4 | exon | Father |
| 5237_3 | 8 | 39351896 | 39497557 | 145662 | gain | IP,QSNP|PCNV | ADAM5P, ADAM3A | exon | Father/Mother |
| 5237_3 | 10 | 67628183 | 67752490 | 124308 | loss | IP,QSNP|PCNV | CTNNA3 | exon | Father |
| 5237_3 | 11 | 70154458 | 70220632 | 66175 | loss | IP,QSNP|PCNV | SHANK2 | exon | *De novo* |
| 5237_3 | 11 | 81181640 | 81194909 | 1327 | loss | IP,QSNP|PCNV | - | - | Father |
| 5237_3 | 14 | 40679974 | 40738084 | 58111 | loss | IP,QSNP|PCNV | - | - | Father/Mother |
| 5237_3 | 14 | 85357100 | 85379917 | 22818 | loss | IP,QSNP|PCNV | - | - | Father |
| 5237_3 | 15 | 28520129 | 28755298 | 23517 | loss | IP,QSNP|PCNV | ARHGAP11B | exon | Mother |
| 5237_3 | 15 | 29807818 | 30303265 | 495448 | gain | IP,QSNP|PCNV | CHRNA7 | exon | Father |
| 5237_3 | 17 | 19443475 | 19478062 | 34588 | loss | IP,QSNP|PCNV | - | - | Father |
| 5237_3 | 17 | 41521621 | 41710573 | 188953 | gain | IP,QSNP|PCNV | KIAA1267 | exon | Father |
| 5237_3 | 19 | 20404485 | 20507201 | 102717 | loss | IP,QSNP|PCNV | - | - | Mother |
| 5237_3 | 22 | 22664948 | 22717669 | 52722 | loss | IP,QSNP|PCNV | GSTTP1, GSTTP2, GSTT1, LOC391322 | exon | Father/Mother |
