# Supplementary material for: Genetic and Functional Analyses of SHANK2 Mutations Suggest a Multiple Hit Model of Autism Spectrum Disorders
Source: PLoS Genet. 2012 Feb 9;8(2):e1002521. doi: 10.1371/journal.pgen.1002521 (PMC3276563; doi:10.1371/journal.pgen.1002521)
Supplement: Table S7 — Distribution of SHANK2 variants affecting conserved or non conserved amino acids. All variants came from this study (26) and from Berkel et al. 2010 (24). *Several variants shared by patients with ASD and controls were identified in both studies. (DOC) [file pgen.1002521.s011.doc]

**Table S7. Distribution of *ProSAP1A/SHANK2* variants affecting conserved or non conserved amino acids.**

|  | Number of variants: Conserved / Total (%) | | | Fisher’s Exact Test, 2-sided | Fisher’s Exact Test, 1-sided |
| --- | --- | --- | --- | --- | --- |
|  | ASD only | ASD & Controls* | Controls only | ASD vs Control | ASD only vs Control only |
| Berkel *et al.* 2010 | 5/6 (83.3%) | 2/8 (25%) | 3/10 (30%) | P = 0.28 | P = 0.059 |
| This Study | 7/9 (77.8%) | 2/8 (25%) | 3/7 (42.9%) | P = 0.31 | P = 0.18 |
| All | 12/15 (80%) | 2/8 (25%) | 6/17 (35.3%) | P = 0.081 | P = 0.013, OR = 6.83, 95% IC = 1.19-53.40 |
