# Supplementary material for: Genetic and Functional Analyses of SHANK2 Mutations Suggest a Multiple Hit Model of Autism Spectrum Disorders
Source: PLoS Genet. 2012 Feb 9;8(2):e1002521. doi: 10.1371/journal.pgen.1002521 (PMC3276563; doi:10.1371/journal.pgen.1002521)
Supplement: Table S8 — Clinical comparison of patients with ASD carrying SHANK2 variants with the rest of the cohort of patients. We used the Wilcoxon test for the continuous traits and the Fisher's exact test (2-sided) for the discontinuous traits. OR is given with 95% confidence interval. OR, odds ratio; P, p-value; ADI-R, Autism Diagnosis Interview revised. (DOC) [file pgen.1002521.s012.doc]

**Table S8. Clinical comparison of patients with ASD carrying *SHANK2* variants with the rest of the cohort of patients.**

|  |  | Patients without SHANK2 variation | Patients with *SHANK2* variations affecting non conserved amino acids | Patients with *SHANK2* variations affecting conserved amino acids |
| --- | --- | --- | --- | --- |
| **Sex** | Male | 314 (77%) | 27 (73%) | 22 (88%) |
|  | Female | 94 (23%) | 10 (27%) | 3 (2.8%) |
|  | P |  | 0.55 | 0.32 |
| **Diagnosis** | Autism | 319 (83.1%) | 26 (74.3%) | 24 (96%) |
|  | Asperger | 42 (10.9%) | 6 (17.1%) | 0 (0%) |
|  | Atypical autism | 23 (6.0%) | 3 (8.6%) | 1 (4%) |
|  | P |  | 0.35 | 0.20 |
| **ADI-R main scores** | Social (cutoff 10) | 21.4 ± 5.8 | 21.1 ± 7.0 | 23.3 ± 5.3 |
|  | P |  | 0.92 | 0.16 |
|  | Verbal communication (cutoff 8) | 17.3 ± 4.6 | 15.5 ± 5.0 | 16.8 ± 5.5 |
|  | P |  | 0.29 | 0.86 |
|  | Non Verbal communication (cutoff 7) | 11.2 ± 3.0 | 10.6 ± 3.9 | 11.7 ± 2.9 |
|  | P |  | 0.70 | 0.49 |
|  | Repetitive behaviors (cutoff 3) | 5.9 ± 2.3 | 6.0 ± 2.2 | 6.0 ± 3.2 |
|  | P |  | 0.67 | 0.93 |
| **Intellectual disability** | IQ>70 | 125 (34.9%) | 12 (37.5%) | **3 (12.5%)** |
|  | IQ<70 | 233 (65.1%) | 20 (62.5%) | **21 (87.5%)** |
|  | P |  | 0.84 | **n=414, P=0.025, OR=3.75 [1.1-20.0]** |
|  | IQ level | 62.8 ± 28.3 | 67.6 ± 33.7 | **48.7 ± 20.9** |
|  | P |  | 0.46 | **n=303, P=0.046** |
| **Seizure** | Absent | 169 (81.3%) | 16 (84.2%) | 15 (93.8%) |
|  | Present | 39 (18.7%) | 3 (15.8%) | 1 (6.25%) |
|  | P |  | 1 | 0.32 |
