# Supplementary material for: Genetic and Functional Analyses of SHANK2 Mutations Suggest a Multiple Hit Model of Autism Spectrum Disorders
Source: PLoS Genet. 2012 Feb 9;8(2):e1002521. doi: 10.1371/journal.pgen.1002521 (PMC3276563; doi:10.1371/journal.pgen.1002521)
Supplement: Table S9 — Primers used for mutation screening. (DOC) [file pgen.1002521.s013.doc]

Table S9. Primers used for mutation screening.

| **Exon** | **Amplicon name** | **Amplicon size (bp)** | **Forward primer (5'-3')** | **Reverse primer (5'-3')** | **Annealing** |
| --- | --- | --- | --- | --- | --- |
| 2 | SHANK2_47-48 | 571 | AGCAACAGCATGATGTGAGG | GGTGGTCACACCAACAAACA | 62°C |
| 3 | SHANK2_45-46 | 574 | CAAACCTCAAGACCCCAGAC | CCTCGGTCTTTCCTTTTTCC | 55°C |
| 4 | SHANK2_43-44 | 633 | TTCAGAGATGCCGCTTTTGA | GGAGACCTCCCAGCTTCTCA | 55°C |
| 5 | SHANK2_58-59 | 964 | CTCATTCACCTCCTCAACAATTC | AGTCAGGAACTTTCCCAGAGC | 55°C |
| 6 | SHANK2_39-40 | 600 | GCTTCACTTCCAAGCTCCAG | AAGGGAGCCTCTTGGACTGT | 62°C |
| 7 | SHANK2_37-38 | 578 | CACTCCTGCTGTGCAGAAAC | ACTGGGTACTCAGGGTGTGG | 61°C |
| 8 | SHANK2_60-61 | 284 | CCGAGTGGCTGGTGTACTTT | GCTGACGTAAGCCAATTTAAGG | 55°C |
| 9 | SHANK2_62-63 | 326 | GCCTCACCTGACAGCATACA | TACAAACACGACCGTGGAGA | 55°C |
| 10 | SHANK2_35-36 | 590 | AAACCTTGCAACAGGGACTG | GGCCCCCACTCATATAGGTT | 55°C |
| 11 | SHANK2_33-34 | 548 | GCTTCCTGCTCAACTTCTGG | GAACTCACAGCTCCCCTCTG | 62°C |
| 12 | SHANK2_31-32 | 555 | GGAATCTCATTCCCAGTGGA | CCTGTCTGGCATCAGGAGTT | 58°C |
| 13 | SHANK2_29-30 | 615 | GGAAGTGCAGTCACACCAGA | GGAAGCTGCCTGTTTTATGC | 55°C |
| 14 | SHANK2_27-28 | 649 | CACGGCAGAATGCTGTTCTA | AAGGTGGCACCAAGTACACC | 55°C |
| 15 | SHANK2_25-26 | 602 | CGGGCCACTTACTTTCTTTG | CCCCCAGGAAAAATGCTTAT | 55°C |
| 16 | SHANK2_23-24 | 538 | GACCACATGCCTCCTCTTTC | CTGGACTAGCCACCTGCAAT | 55°C |
| 17 + 18 | SHANK2_21-22 | 633 | GCACCTTTGCTTCCAAAAAG | GGATCTTGCCATATCCCTGA | 55°C + tpQ 5% |
| 19 | SHANK2_19-20 | 517 | GGGGCTGCTTACCTTTATCC | TAAGCACACCTGCACAATCC | 55°C |
| 20 | SHANK2_56-57 | 441 | TTTGCGACACATTTGAGACTTC | CTGAGAACTCCTCCCTACTTTGG | 55°C |
| 21 | SHANK2_13-14 | 631 | TTCTATGTCCATGCCTCGTG | GGGTCCTCAGCCAAGTATGA | 55°C |
| 22 | SHANK2_11-12 | 523 | AGCTGCAGCGAATAGGAAAG | TCATGCCACTCAGATGTTCC | 55°C |
| 23 | SHANK2_9-10 | 587 | ATTGCAAAGATGGCTCGTTC | CTGTTGCAGGCAAAGGTACA | 55°C |
| 24 | SHANK2_7-8 | 998 | GACTAACCACTCGGCATCGT | GCTGTCCTCGTCAGCAAAAT | 55°C |
| 24 | SHANK2_5-6 | 993 | GACCTGGGGGATGAGGATGT | GTCTGAGAGAGCCGGGACAG | 55°C |
| 24 | SHANK2_3-4 | 961 | ATGGAAGAGGCGGTGATTTT | CCCACACAGGCTTTACGAAT | 55°C |
| 25 | SHANK2_1-2 | 784 | GACCTGCCTTGTGATGGTTT | GTCTGAAGACCCAGCCATGT | 55°C |
