# Supplementary material for: Genetic and Functional Analyses of SHANK2 Mutations Suggest a Multiple Hit Model of Autism Spectrum Disorders
Source: PLoS Genet. 2012 Feb 9;8(2):e1002521. doi: 10.1371/journal.pgen.1002521 (PMC3276563; doi:10.1371/journal.pgen.1002521)
Supplement: Table S10 — Primers used for in vitro mutagenesis. (DOC) [file pgen.1002521.s014.doc]

**Table S10. Primers used for *in* *vitro* mutagenesis.**

| **Mutation** | **Forward primer (5'-3')** | **Reverse primer (5'-3')** |
| --- | --- | --- |
| ProSAP1A_S557N | agggtcaaagttctgaacatcggcgagggcg | cgccctcgccgatgttcagaactttgaccct |
| ProSAP1A_R569H | aaggcagcgcccatggccacatcgg | ccgatgtggccatgggcgctgcctt |
| ProSAP1A_L629P | gacaagacggtggtcccgcagaagaaagacaac | gttgtctttcttctgcgggaccaccgtcttgtc |
| ProSAP1A_V717F | ctcgtccttaaggtcttcacggtgaccagga | tcctggtcaccgtgaagaccttaaggacgag |
| ProSAP1A_A729T | gaatctagaccctgatgatacaaccagaaagaaagctcc | ggagctttctttctggttgtatcatcagggtctagattc |
| ProSAP1A_K780Q | gtcccagcctcccagccctccagga | tcctggagggctgggaggctgggac |
| ProSAP1A_R818H | ctccgtgtacgagcaccaagggattgctg | cagcaatcccttggtgctcgtacacggag |
| ProSAP1A_A822T | gagcgccaagggattactgtaatgacgccc | gggcgtcattacagtaatcccttggcgctc |
| ProSAP1A_V823M | cgccaagggattgctatgatgacgcccacggtc | gaccgtgggcgtcatcatagcaatcccttggcg |
| ProSAP1A_Y967C | tcagaagacgtctgcagccgcagcccc | ggggctgcggctgcagacgtcttctga |
| ProSAP1A_G1170R | GCTCAGGGGGAGGCTAGGGGACCC | GGGTCCCCTAGCCTCCCCCTGAGC |
| ProSAP1A_R1290W | cctgagcaaggactggagggctgacga | tcgtcagccctccagtccttgctcagg |
| ProSAP1A_Q1308R | gacactgcccagcggaagtcagccggc | gccggctgacttccgctgggcagtgtc |
| ProSAP1A_D1535N | ggatacttgcacagtctatgcaaacgggcaagcct | aggcttgcccgtttgcatagactgtgcaagtatcc |
| ProSAP1A_P1586L | ccgcccccgctcggcagtgcc | ggcactgccgagcgggggcgg |
| ProSAP1A_L1722P | ggaattgagcaaagagattccgcccacccctccg | cggaggggtgggcggaatctctttgctcaattcc |
