# Supplementary material for: Genetic and Functional Analyses of SHANK2 Mutations Suggest a Multiple Hit Model of Autism Spectrum Disorders
Source: PLoS Genet. 2012 Feb 9;8(2):e1002521. doi: 10.1371/journal.pgen.1002521 (PMC3276563; doi:10.1371/journal.pgen.1002521)
Supplement: Table S11 — Primers used for mRNA analysis of SHANK2 isoforms. * Primers were used for relative quantification study of SHANK2E isoform. The other primers were used for RT-PCR analysis of each SHANK2 isoform. (DOC) [file pgen.1002521.s015.doc]

**Table S11. Primers used for mRNA analysis of *SHANK2*** isoforms.

| **Exon** | **Amplicon name** | **Amplicon size (bp)** | **Forward primer (5'-3')** | **Reverse primer (5'-3')** | **Annealing** |
| --- | --- | --- | --- | --- | --- |
| E6-E10 | SHANK2E | 445 | gatgaccgccctacacaaag | cagctcaaagttgcctgcta | 58°C |
| E6-E7 | SHANK2E* | 81 | AGTTGCCCTGAAGACCCTTT | GTGTGATACAGCGGGGTGAG | 60°C |
| E1b-E11 | ProSAP1A | 305 | gaagtctttgttaaatgccttcac | CCAGCCTGTTGAGCGATG | 57°C |
| E1c-E16 | ProSAP1 | 252 | GTGATGATGACGGGCTACAA | GAAGTCCCCGGTCCTTAGTC | 55°C |
| E15-E17 | All isoforms or PDZ domain | 333 | TTGAGGAGAAGACGGTGGTC | TTTCTTCCTGGCGGTGTCG | 58°C |
| E18-E21 | Splicing E19 & E20 | 154 | ACCGACCACAGCCCTCAC | TTCCACAGCCATGTTCTCAG | 55°C |
| E21-22 | AF141901 | 456 | CTGAGAACATGGCTGTGGAA | TGGGCTTCAAGATGACAGAA | 55°C |
| E21-24 | Splicing E23 | 404 | CTGAGAACATGGCTGTGGAA | CGTCCCGTAGACTCTTGGAG | 55°C |
