# Supplementary material for: Genetic and Functional Analyses of SHANK2 Mutations Suggest a Multiple Hit Model of Autism Spectrum Disorders
Source: PLoS Genet. 2012 Feb 9;8(2):e1002521. doi: 10.1371/journal.pgen.1002521 (PMC3276563; doi:10.1371/journal.pgen.1002521)
Supplement: Table S12 — Primers used for CNV validation. (DOC) [file pgen.1002521.s016.doc]

**Table S12. Primers used for CNV validation.**

| **Exon** | **UPL probe ROCHE** | **Forward primer (5'-3')** | **Reverse primer (5'-3')** |
| --- | --- | --- | --- |
| E4 | #62 (cat. no. 04688619001) | cgtcctccttgttatcttgttgt | ggccagtctgcctgtacatc |
| E5 | #22 (cat. no. 04686969001) | ttgtttttccttctagaccaatctg | cctcggtccagcatcttg |
| E6 | #22 (cat. no. 04686969001) | cgactctgtggaggtcatca | catctttggcacggaagtc |
| E15 | #27 (cat. no. 04687582001) | ctcgctgcctcctttcct | catcatcatagccaattaatcacc |
| E16 | #33 (cat. no. 04687663001) | caggcttgtaaccaggtgct | cggtgttggtgtgaattcttc |
| E17 | #55 (cat. no. 04688520001) | ccgctggctctcactgat | tggccgactttgacaacat |
